# Reconnoitering the Therapeutic Role of Curcumin in Disease Prevention and Treatment: Lessons Learnt and Future Directions

**DOI:** 10.3390/metabo12070639

**Published:** 2022-07-12

**Authors:** Bala Mohan Sivani, Mahmoud Azzeh, Rajashree Patnaik, Anca Pantea Stoian, Manfredi Rizzo, Yajnavalka Banerjee

**Affiliations:** 1 Banerjee Research Group, College of Medicine and Health Sciences, Mohammed Bin Rashid University of Medicine and Health Sciences (MBRU), Dubai 505055, United Arab Emirates; sivanimohan0308@gmail.com (B.M.S.); mahmoud.azzeh@students.mbru.ac.ae (M.A.); rajashree.patnaik@mbru.ac.ae (R.P.); 2 Department of Diabetes, Nutrition and Metabolic Diseases, Carol Davila University of Medicine and Pharmacy, 020022 Bucharest, Romania; ancastoian@yahoo.com; 3 Department of Health Promotion, Mother and Child Care, Internal Medicine and Medical Specialties (Promise), University of Palermo, 90128 Palermo, Italy; manfredi.rizzo@unipa.it; 4 Centre for Medical Education, University of Dundee, Dundee DD1 4HN, UK

**Keywords:** *Curcuma longa*, nutraceutical, curcumin, antiinflammation, antimicrobial, antioxidant, anticancer, antiviral, SARS-CoV-2, turmeric, functional food, clinical trial

## Abstract

Turmeric is a plant with a very long history of medicinal use across different cultures. Curcumin is the active part of turmeric, which has exhibited various beneficial physiological and pharmacological effects. This review aims to critically appraise the corpus of literature associated with the above pharmacological properties of curcumin, with a specific focus on antioxidant, anti-inflammatory, anticancer and antimicrobial properties. We have also reviewed the different extraction strategies currently in practice, highlighting the strengths and drawbacks of each technique. Further, our review also summarizes the clinical trials that have been conducted with curcumin, which will allow the reader to get a quick insight into the disease/patient population of interest with the outcome that was investigated. Lastly, we have also highlighted the research areas that need to be further scrutinized to better grasp curcumin’s beneficial physiological and medicinal properties, which can then be translated to facilitate the design of better bioactive therapeutic leads.

## 1. Introduction

In recent times, there has been an increased impetus to reconnoitre the medicinal properties of food. Case in point, Rao et al., in a recent exploratory study, assessed the safety and prospective efficacy of *Nigella sativa* and fenugreek seed-supplemented chapattis (unleavened flatbread originating from the Indian subcontinent) in obese and type–2 diabetic subjects to demonstrate that the consumption of chapattis combined with *N. sativa*/fenugreek triggered a significant clinical improvement in obesity and diabetes. However, the other key highlight of this study was the long-term compliance of 100% [1]. Although the compliance regarding a clinical trial may vary from the compliance during the regular use of a food product in the community, the adherence to the dietary intervention in this study is a reason for optimism, whereby treatment for chronic diseases can be effectively delivered through food. Hence, this school of thought has coined the term “Functional Foods”.

The term “Functional foods (FFs)” was first created and defined in the 1980s by the Ministry of Health and Welfare of Japan when they established a regulatory system for foods that possess possible health benefits [2]. FFs were defined as foods that keep constructive effects on target functions in the physiological milieu of humans beyond nutritional effects, aiming for health promotion and wellbeing and/or the reduction of chronic diseases. With time, FFs have attained popularity, with a market value of USD 1.7 billion as of today. Further to the above, FFs have given way to the term “nutraceutical”.

The term “nutraceutical” was coined from “nutrition” and “pharmaceutical” in 1989 by the founder and chairman of the Foundation for Innovation in Medicine (FIM) situated in Cranford, NJ, Stephen DeFelice, MD [3]. According to DeFelice, a nutraceutical is “a food (or part of a food) that provides medical or health benefits, including the prevention and/or treatment of a disease” [3]. In other words, the term “nutraceutical” implies the pharmaceutical formulation of the bioactive compound, whose concentration often shifts from its natural concentration in food. No regulatory evaluations and no toxicological assessments are required. Therefore, one can say that when FF aids in the prevention and/or treatment of disease(s) and/or disorder(s) other than anaemia, it is called a nutraceutical [4]. However, an important point to remember is that the term nutraceutical, as commonly used in marketing, has no regulatory definition. Hence, broadly, nutraceuticals are foods or parts of food playing an important role in modifying and maintaining the customary physiological function that maintains healthy human beings. Case in point, allyl sulphur compounds in garlic, quercetin in berries, EPA [5] (*Eicosapentaenoic acid*) and DHA (*Docosahexaenoic acid*) in fish oils, curcumin in turmeric, ginsenosides from ginseng roots and polyphenolic catechins in green tea are some of the nutraceuticals that have been studied extensively [6].

There is an increased interest in the niche of nutraceutical research because nutraceutical(s) are generally associated with lesser side effects. For example, oleocanthal (OC), present in significantly high concentrations in extra-virgin olive oil (EVOO), is a structural analogue of ibuprofen and, like ibuprofen, mediates anti-inflammatory properties by the inhibition of cyclooxygenase (COX) enzymes in the prostaglandin biosynthesis pathway [7,8]. Similarly, lycopene, which is a tetraterpene compound abundant in tomato and tomato-based products, is essentially recognized as a potent antioxidant and a non-pro-vitamin A carotenoid. Lycopene has been shown to ameliorate cancer insurgences, diabetes mellitus, cardiac complications, oxidative stress-mediated malfunctions, inflammatory events, skin and bone diseases and hepatic, neural and reproductive disorders [9]. Likewise, reserveratol, which is an activator of SIRT1, one of the mammalian forms of the sirtuin family of proteins, mediates its beneficial effects on metabolism, stress resistance, cell survival, cellular senescence, inflammation–immune function, endothelial functions and circadian rhythms [10]. Curcumin isolated from the turmeric plant is one of those single molecules that have been appraised extensively in numerous in vitro and in vivo studies. However, few of the effects observed in such studies have been replicated efficiently in the numerous clinical trials that have been conducted with curcumin (see below for details). This raises the question, “Should we continue to explore the beneficial properties of curcumin, which some have christened as “The Golden Spice”?” Furthermore, “is there a definite mechanism by which curcumin mediates its effect?” In this review, we tackle these aspects of curcumin structure-function and address some of the surrounding controversies.

**Figure 1:**
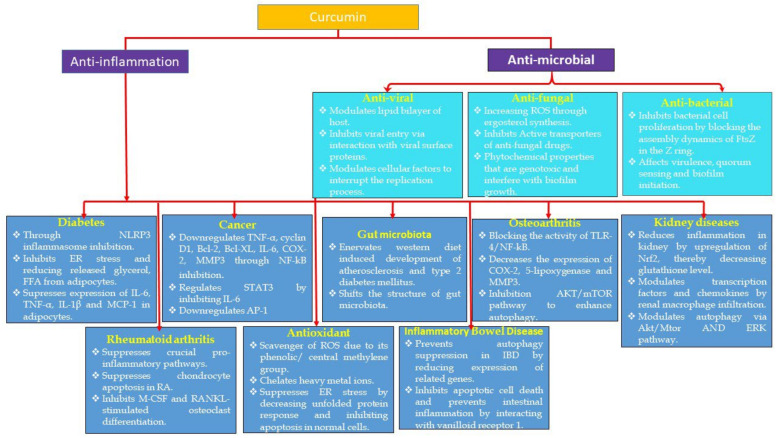
A flow diagram summarizing the plausible mechanism of action of curcumin.

Turmeric is a plant has a very long history of medicinal use, especially in Indian culture, dating back nearly 4000 years. It is used as a culinary spice and has immense religious significance. In India, turmeric is colloquially referred to as “Haldi”, which, when literally translated, denotes the color yellow and is responsible for the precise yellow color of the traditional Indian curry. Over time, the use of turmeric also reached other parts of the globe. It possibly reached China by 700 AD, East Africa by 800 AD, West Africa by 1200 AD and Jamaica in the eighteenth century. In 1280, Marco Polo alluded to this spice, marvelling at a vegetable that displayed virtues like saffron. According to Sanskrit medical discourses and the alternative medicinal disciplines of the Ayurveda and Unani systems, turmeric has a long history of therapeutic use in South Asia. In fact, Susruta’s Ayurvedic Compendium, dating back to 250 BC, endorses the use of an ointment containing turmeric to relieve the effects of poisoned food. The medicinal property of turmeric is attributed to the bioactive main natural polyphenol compound curcumin, (1,7-bis(4-hydroxy-3-methoxyphenyl)-1,6-heptadiene-3,5-dione), also called diferuloylmethane. Curcumin mediates a plethora of beneficial physiological effects. Due to its ability to interact with various molecular targets, curcumin is one of the most interesting pleiotropic nutraceuticals [11]. The antioxidant, anti-inflammatory, anticancer and antimicrobial properties of curcumin have been extensively researched and appraised in different in vitro and in vivo experimental models. Figure 1 gives an overview of the pharmacological activities of curcumin, which will be further discussed in detail in the manuscript.

This review aims to critically appraise the corpus of literature associated with the above pharmacological properties of curcumin, with a specific focus (Figure 1) on identifying the gaps that need to be addressed to obtain a better insight into the molecular mechanism through which curcumin mediates these advantageous effects. Lastly, we have also highlighted the research areas that need to be further scrutinized to better grasp the beneficial physiological and medicinal properties of curcumin, which can then be translated to facilitate the design of better bioactive therapeutic leads.

### 1.1. Source of Curcumin

**Figure 2:**
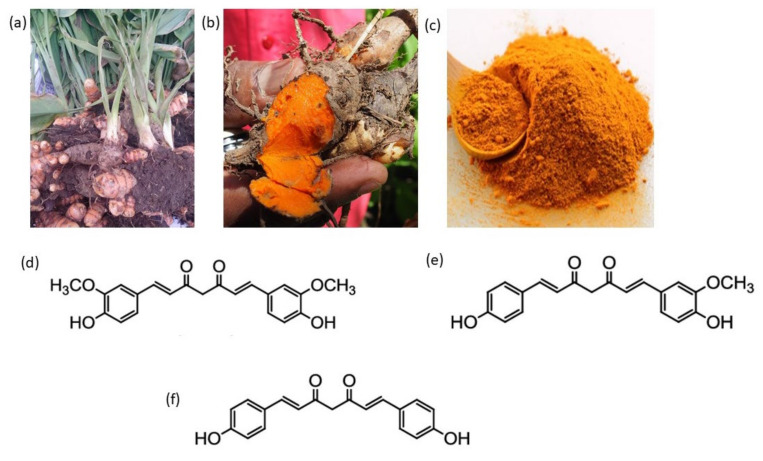
(**a**) The turmeric plant, (**b**) The turmeric rhizome with a yellow-orange interior, (**c**) The powdered form of turmeric, (**d**) The chemical structure of curcumin. (**e**) Demethoxycurcumin and (**f**) Bis-demethoxycurcumin.

Curcumin is the principal curcuminoid of the turmeric plant *Curcuma longa*. Curcumin was discovered around two centuries ago when Vogel and Pelletier reported the isolation of “yellow colouring-matter” from the rhizomes of *Curcuma longa* (turmeric) and named it curcumin [12]. The turmeric plant, *Curcuma longa*, is a rhizomatous herbaceous perennial plant belonging to the ginger family Zingiberaceae, which is native to tropical South Asia (Figure 2a–c) [13]. As many as 133 species of *Curcuma* have been identified worldwide [14]. Turmeric is derived from the mature tuberous rhizome of *C. longa*. Once the rhizomes mature underground (beneath the foliage), they become yellowish-brown with a dull orange interior (Figure 2b) [15]. This yellowish color is because of the presence of curcuminoids, which are natural polyphenol compounds classified into three diarylheptanoids (diferuloylmethane derivatives): curcumin (77%), demethoxycurcumin (17%), bisdemethoxycurcumin (3–6%) (Figure 2d-f) and other less abundant secondary metabolites.

### 1.2. Chemistry and Bioavailability of Curcumin

Curcumin has been identified as 1,6-heptadiene-3,5-dione-1,7-bis(4-hydroxy-3-methoxyphenyl)-(1E,6E) or diferuloylmethane (Figure 2d) [16]. It is an orange-yellow crystalline powder that is relatively insoluble in water (limiting its medicinal use in humans when taken orally or injected) and ether but soluble in acetone, ethanol and acetic acid dimethylsulfoxide [17]. It has a melting point of 183 °C, a molecular formula of C_21_H_20_O_6_ and a molecular weight of 368.37 g/mol [18]. Spectrophotometrically, the maximum absorption (lmax) of curcumin in methanol occurs at 430 nm, and, in acetone, it occurs at 415–420 nm [19]. The powder gives a brownish-red color with alkali and a light-yellow color with acids [20]. Curcumin exists in enolic and β-diketonic forms. The fact that curcumin in solutions exists primarily in its enolic form has been the key to the radical scavenging ability of curcumin [21]. It is stable in acidic pH, but in neutral and basic pH, it gets degraded to ferulic acid and feruloylmethane. Curcumin rapidly degrades when placed in a phosphate buffer solution at a pH of 7.2, whereas in ascorbic acid, N-acetylcysteine and glutathione, it does not degrade, which explains the oxidative mechanism of these anti-oxidative agents [22]. Curcumin is poorly absorbed in the gastrointestinal tract (one of the key hurdles to increasing the bioavailability of curcumin). Case in point, a poor absorption from the gut was observed in rats after the oral administration of curcumin at a dose of 1 g/kg bw, which led to 75% fecal excretion with traces in the urine, and the concentration of curcumin was below 5 ug/mL in the plasma [23,24]. The oral administration of radio-labelled curcumin at a 0.6 mg/kg dose in rats resulted in 89% excretion in feces and 6% excretion in the bile after 72 h. At the same dose, when administered peritoneally, 73% fecal excretion was observed, and 11% excretion was observed in the bile [25]. A slightly better absorption rate of 60% was seen upon 400 mg of curcumin being administered orally, and 40% faecal excretion was observed over a period of 5 days [26].

### 1.3. Extraction of Curcumin

Several extraction strategies have been availed for the isolation of curcumin. Conventional methods such as solvent extraction, Soxhlet extraction and hydro/steam distillation are time-consuming, are not eco-friendly and have low efficiencies. In this review, we have not touched upon these techniques. The readers are referred to the excellent review of Zhang et al. for details, if interested [27]. Novel extraction techniques (summarized in Table 1, with the associated advantages and drawbacks of these techniques) have been effectively strategized to maximize the extraction efficiency, decrease the use of toxic solvents and concomitantly be cost-effective.

## 2. Methods

Relevant publications were searched for in PubMed (https://pubmed.ncbi.nlm.nih.gov (accessed on 29 April 2022) and Google Scholar (https://scholar.google.com (accessed on 29 April 2022), using the various names of curcumin and its related functions such as antimicrobial, antiinflammation, anti-fungal, antibacterial, rheumatoid arthritis, cancer, diabetes, inflammatory bowel disease and gut microbiota as keywords. The lead author (BMS) and the corresponding author (YB) finalized the keywords, which were vetted and agreed upon by all the authors. The search was conducted independently by two authors (BMS and MA). Overlaps were eliminated, and the final list of selected articles was agreed upon and vetted by all participating authors. The search was restricted to articles published only in English. The images are shown in Figure 2a The turmeric plant, (b) the turmeric rhizome with a yellow-orange interior, (c) the powdered form of turmeric and (d) the chemical structure of curcumin were adopted with minor modifications from the Wiki commons [33,34].

## 3. Anti-Inflammatory Properties of Curcumin

**Figure 3:**
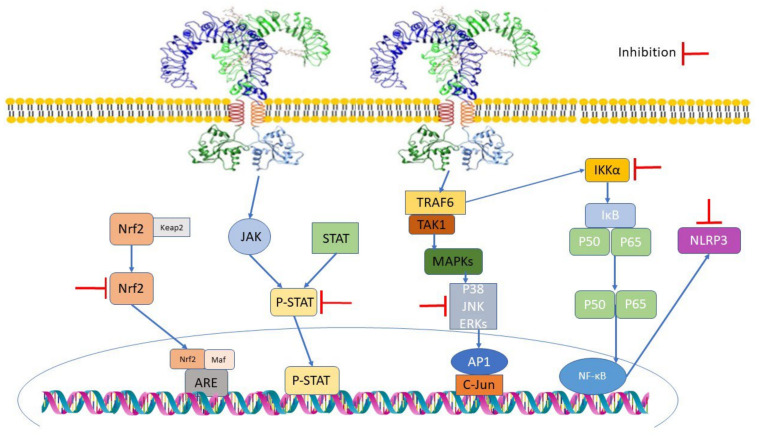
Role of curcumin in the inflammatory signaling pathway.

Inflammation is a response to tissue damage caused by oxidative stress, pathogens, chemicals or radiation and triggers repair. Chronic inflammation lasts from several months to years due to tissue invasion by inflammatory cytokines and growth factors. Curcumin shows an anti-inflammatory property by interacting with Toll-like receptors (TLRs), which play a key role in innate immunity [35]. Upon binding, it regulates the production of inflammatory mediators such as Mitogen-activated protein kinases (MAPK), Activator Protein 1 (AP-1) and Nuclear Factor Kappa-B (NF-κB) [36]. The Janus kinase/Signal transducer and activator of the transcription (JAK/STAT) signaling pathway has been one of the main targets to treat inflammatory diseases such as rheumatoid arthritis and inflammatory bowel diseases. Curcumin has also been proven to regulate JAK/STAT signaling. Another way to decrease inflammation is by regulating inflammatory mediators. Case in point, curcumin has decreased the level of mediators such as Interleukin-1 (IL-1), IL-17, IL-27, IL-6, IL-8, IL-1β [37], Tumor necrosis factor-α, Monocyte chemotactic protein-1 (MCP-1) and Inducible nitric oxide synthase (iNOS). Nuclear factor erythroid 2 p45-related factor (Nrf2) overactivation is seen in neoplasms [38] and has also been linked with insulin resistance in diabetes. Curcumin has suppressed proteins such as Keap1, which interacts with Nrf2, thereby regulating its overexpression. In Figure 3, we have summarized curcumin’s anti-inflammatory property via the inhibition of signalling pathways.

One of the most important complexes that participate in inflammation is the inflammasome. Among the various inflammasomes, the NOD-like receptor pyrin domain containing 3 (NLRP3) detects the products of damaged cells and triggers an immune response. It mainly involves two plausible mechanisms:

(1) Inflammatory bacterial products such as lipopolysaccharide(LPS) activate the NF-κB pathway to activate NLRP3, leading to Pro-Interleukin-1β (pro-IL-1β) synthesis.
(2) Stimuli such as nigericin, aluminium crystal and monosodium urate crystal lead to NLRP3 activation, subsequently leading to the activation of caspase-1 along with the promotion of proinflammatory cytokines such as IL-1B and IL-18 [39].

NK-κB plays a vital role in aggregating NLRP3 components to form an active NLRP3 inflammasome. Curcumin has been shown to suppress the activation of the NLRP3 inflammasome and IL-1B secretion by regulating the NK-κB pathway [40]. Additionally, curcumin also inhibits the NLRP3 inflammasome by preventing Ca^2+^ influx and attenuating K^+^ efflux, thereby disrupting the formation of NLRP3 components [41]. Therefore, NLRP3 is one of the best targets by which curcumin can treat various inflammatory diseases. The key inflammatory diseases for which the beneficial effects of curcumin have been extensively investigated/appraised are depicted below.

### 3.1. Rheumatoid Arthritis (RA)

RA is a chronic inflammatory disease affecting the joints and causing irreversible bone, synovium and cartilage degradation, reduced mobility and discomfort. Curcumin has been found to suppress pro-inflammatory pathways crucial in the development of RA. A study by Wang et al. and Murakami et al. demonstrated that curcumin increased macrophage apoptosis and decreased the level of IKBα, thereby reducing the expression of COX-2 and inhibiting the activation of NF-κB [42,43]. Curcumin has also been shown to inhibit lymphocyte proliferation and decrease IL-4 and IL-5 levels and the granulocyte-macrophage colony-stimulating factor in lymphocytes [44]. Moreover, curcumin augments the activity of anti-inflammatory IL-10, inhibits BAFF (B cell-activating factor) expression and suppresses STAT1 signaling [45]. An in vivo study with curcumin by da Silva et al. revealed decreased infiltration and neutrophil activation, which prevents the migration of neutrophils from the blood to inflamed joints, acting as a proapoptotic agent in RA treatment. Curcumin also increases the surface expression of the cluster of differentiation (CD) 16^+^ and CD 56^dim^ in natural killer cells, proving its immunostimulatory activity [46]. Experiments conducted by Moon et al. [47] and Dai et al. [48] explained the anti-inflammatory property of curcumin in synovial fibroblasts, where it suppresses COX-2 (this blocks the synthesis of prostaglandin E2), reducing synovial cell hyperplasia via the Mtor pathway and downregulating various NK-κB complexes, IL-1β and TNF-α [47,49].

Chondrocyte apoptosis seen in RA has been responsible for joint cartilage damage. In a study, curcumin inhibited IL-1β-induced IKBα phosphorylation and the activation of caspase-3 and COX-2 in chondrocytes isolated from cartilage (this might support cartilage regeneration in RA) and suppressed apoptosis in these chondrocytes [50]. In in vivo RA models, curcumin has been shown to decrease IL-1β, IL-18RA, IL-6, IL-18, TNF-α, IFN-gam, MMP3 [51] and IL-17 [42]. Bone degradation in RA by osteoclasts has been investigated by Shang et al., employing peripheral blood mononuclear cells (PBMCs) obtained from patients with RA with different concentrations of curcumin (2.5–10 µM) for 48 h. The results from this study demonstrated that curcumin inhibited M-CSF and RANKL-stimulated osteoclast differentiation via the suppression of ERK1/2, p38 and JNK activation. Another study evaluated curcumin’s capacity for inhibiting human osteoclastogenesis. Curcumin concentrations in the range of 1–10 µM inhibited osteoclast differentiation and bone-resorption, indicating that curcumin could be a potential therapeutic leading to managing bone deterioration in RA. It has also been reported that curcumin supplementation (500 mg for 8 weeks) [52] or curcumin nanomicelle administration (40 mg, 3 times a day over a period of 12 weeks) in RA patients tend to decrease the tenderness and swelling of the joints [53].

### 3.2. Osteoarthritis (OA)

Osteoarthritis (OA) is one of the leading causes of morbidity and disability worldwide. The prevalence of OA is projected to increase in the future [54,55]. The disease’s exact pathophysiology is not yet completely understood. Nonetheless, biomechanical (wear and tear), inflammatory and metabolic factors have been implicated in inducing the sterile inflammation and catabolism of the cartilage of the joint [56,57,58]. To date, there is no effective treatment to prevent or halt the progression of the disease that has been discovered [59]. The available pharmacological interventions, including non-steroidal anti-inflammatory drugs (NSAIDs) and acetaminophen, target the symptomatic treatment of pain [60]. Nonetheless, the prolonged use of NSAIDs is associated with significant cardiovascular, renal and gastrointestinal adverse events [61,62,63]. Curcumin emerged as a safe alternative for pain symptom relief and has been studied in preclinical and clinical trials.

In several preclinical studies, curcumin has shown positive effects on the reduction of inflammatory and catabolic markers in OA rat models [64,65]. Yan et al. have examined the effects of intra-articular curcumin injections in OA-induced rat knee models. Inflammatory markers in OA, including the Toll-like receptor (TLR)- 4 and its downstream pathway including NF-κB, IL-1β and TNF-α, were reduced significantly [64]. Additionally, curcumin preserved cartilage thickness and reduced the number of apoptotic chondrocytes in microscopic studies [64]. Zhang et al. showed similar findings with an intraperitoneal injection of curcumin [66,67]. The oral curcumin effects on rat OA models showed similar findings of decreased serum levels of cyclooxygenase-2 (COX-2) and 5-lipoxygenase, which are responsible for pain and inflammation. Matrix metalloproteinase-3 (MMP-3) proteins, which are highly expressed in osteoarthritic tissues and are responsible for breaking down cartilage by degrading the extracellular matrix in osteoarthritic joints, were also reduced [68]. Additional in vitro studies revealed the decreased activation of proapoptotic protein caspase-1 and the decreased expression of MMP-3 and displayed a dose-dependent inverse relationship between curcumin and MMP-3 levels [65]

Reduced autophagy and increased apoptosis have been indicated in the pathophysiology of OA [69,70]. In vivo experiments revealed that curcumin admiration decreased caspase-3 and Bax/Bcl2 levels, reducing apoptosis, while autophagic activity was high through the increased expression levels of light chain-3 (LC-3) [66]. Additionally, in vitro, mechanistic studies revealed the inhibition of the AKT/mTOR pathway by curcumin, which resulted in reduced apoptosis and enhanced autophagy [66,71].

Clinical studies of oral curcumin showed promising results in alleviating OA symptoms [72]. Several studies have shown benefits on the pain and functional scores of OA after administering oral curcumin alone or as an adjunct [73,74,75,76,77]. Previous lab studies revealed the synergism of COX-2 inhibitors and curcumin by reducing the expression of the enzyme and reducing prostaglandin E2 levels, which has translated in clinical trials into reducing pain symptoms and improving functional outcomes [78,79]. Additionally, Shep et al. showed that patients using NSAIDs and curcumin reported reduced GI pain as compared to patients receiving NSAIDs alone [77]. Patients on curcumin were able to decrease their daily dosage of NSAIDs owing to the analgesic effect of curcumin. Kuptniratsaikul et al. compared ibuprofen against ibuprofen and showed similar outcomes on the Western Ontario and McMaster Universities Osteoarthritis Index (WOMAC) [76]. An exploratory trial has shown decreased Coll2-1, a novel OA marker in patients’ serum, after administering curcumin [75]. A recent systematic review and metanalysis by Paultre et al. concluded that heterogenous curcumin is safe and beneficial in terms of the pain and function of patients with knee OA [72].

### 3.3. Cancer

Inflammation causes an increase in the production of pro-inflammatory molecules such as cytokines, reactive oxygen species (ROS), cyclooxygenase (COX)-2, transcription factors such as NF-κB, protein kinases B, activator protein 1(AP-1) and the signal transducer and activator of transcription 3(STAT3), leading to the initiation and development of cancer [80]. Curcumin shows a similar activity as that seen in RA, where it suppresses NF-κB activity by inhibiting IκB. It downregulates the expression of inflammatory genes such as TNF-α[81] and downregulates cyclin D1, Bcl-2, Bcl-xL, IL-6, COX-2 and MMP9 through NF-κB inhibition [82]. Curcumin has also been shown to downregulate AP-1 (known to be related to anti-apoptotic genes). In addition, Curcumin is directly or indirectly related to the regulation of STAT3 (a protein that promotes oncogenesis) by inhibiting IL-6 [83]. The anticancer effects of curcumin observed in different cancer models are summarized in Table 2.

### 3.4. Diabetes

Inflammation plays a pivotal role in diabetes. In fact, a review of the possible mechanisms that drive the metabolic pattern in Type 1 Diabetes and Type 2 Diabetes (T1D and T2D) and the involved inflammatory pathways indicates that the effective management of diabetes requires the modulation of the inflammatory pathways. In line with this, in this review, we will critically appraise the different cell and animal models that have been employed in investigating the anti-diabetic effects of curcumin, identifying the key results obtained in these studies. First, stress-causing factors such as obesity stimulate NK-κB activity and cause insulin resistance in adipose tissue, the liver and leukocytes. Second, curcumin supplementation has significantly reduced the NLRP3 inflammasome by inhibiting its activation, downregulating the NK-κB pathway and thereby reducing the caspase-1 activation and IL-1B secretion. Another anti-inflammatory activity of curcumin is the inhibition of ER stress in adipocytes by preventing the phosphorylation of phospho-inositol-requiring kinase 1(p-IRE1) and phospho-eukaryotic Initiation Factor 2 (p-eIF2) [163]. It also reduced the glycerol level and FFA released from adipose tissues [164]. The third mechanism is the inhibition of the pro-inflammatory NF-κB signaling pathway activation. Therefore, curcumin has shown beneficial anti-inflammatory effects by suppressing the expression of IL-6, TNFα, IL-1β [165] and MCP-1 from adipocytes [166] by inhibiting the recruitment of macrophages in adipose tissues and inhibiting NLRP3 inflammasome activity [40]. The effect of curcumin on diabetes has been summarized in Table 3.

In experimental models, chemicals such as streptozotocin (STZ) and alloxan have been used to induce diabetes. In mice, low doses of STZ (i.e., 40 mg/kg intraperitoneally injected for 5 consecutive days) have closely resembled human T1DM, with chronic pancreatic islet inflammation, insulitis and insulin deficiency. In rats, a single dose of STZ (i.e., 65 mg/kg) is required to generate T1DM, and high doses of STZ cause the toxin-induced necrosis of B cells, hypoglycemia and cell death. For T2DM, the exposure to a high-fat diet (60% fat by caloric content) followed by a moderate dose of STZ has resulted in hyperglycemia and insulin resistance [167]. None of the above models mimic human T1DM and T2DM. Therefore, the choice of model depends on the aim of the study. Challenges such as the regulation of STZ specificity and toxicity, the careful monitoring of diets and other factors and the ethics involved in the use of animal models should be kept in mind for the appropriate induction of diabetes using STZ [168].

Alloxan has been effectively administered at 170–200 mg/kg BW intraperitoneally to induce diabetes in animal models. However, alloxan-induced hyperglycemia is not sufficiently stable for the proper evaluation of antidiabetic compounds. It induces diabetes by a mechanism characterized by reactive oxygen species toxicity, ketosis and a high mortality rate. Instability, poor diabetogenicity, easy auto-reversal and the route and speed of administration are the factors to be considered to improve the use of alloxan as a diabetogenic drug [169].

**Table 3:** Summary of the anti-diabetic role of curcumin and the mechanism of action.

Model	Conc. of Curcumin	Increase	Decrease	No. of Mice/Rats Used	Route of Administration	Reference
Albino Wistar rats with Streptozotocin-induced diabetes	0.5% of diet; 8 weeks	ATPase activity, PUFA/SFA ratio	Phospholipid, triglyceride, kidney weight, renal lesion progression, renal damage, urine ALT and AST, kidney alkaline and acid phosphatase, glucose-6- phosphatase	48	Intraperitoneal	[170]
Albino Wistar rats with Streptozotocin-induced diabetes	300 mg/kg b.w./day for 8 weeks	Creatinine, kidney SOD activity, kidney catalase activity	Glucose, total cholesterol, triglyceride, urea, body weight, kidney lipid peroxidation	10	Intraperitoneal	[171]
Wistar Rats with Streptozotocin-induced diabetes	80 mg/kg b.w./day; 45 days	Insulin, SOD, catalase, GPx activity, glutathione-S-transferase	Glucose, lipid peroxidation, TBARS, H_2_O_2_	24	Intraperitoneal	[172]
Sprague–Dawley rats with Streptozotocin-induced diabetes	15 and 30 mg/kg b.w./day; 2 weeks	Creatinine clearance, SOD activity, catalase activity	Glucose, creatinine, renal changes, oxidative stress, urine albumin, proteinuria, lipid peroxidation, MDA	N/A	Intraperitoneal	[173]
Wistar-NIN rats with Streptozotocin-induced diabetes	0.01% curcumin; 8 weeks	SOD activity, pancreas catalase activity	Glucose, insulin, TBARS, pancreas SOD activity, glutathione-S-transferase activity	32	Intraperitoneal	[174]
Sprague–Dawley rats with Streptozotocin induced type 1 diabetes	50 mg/kg b.w./day; 6 weeks	Albumin, acetyl-histone H3, phospho-histone H3	Urea, creatinine, HSP-27 protein, p38 protein	12	Intraperitoneal	[175]
C57/BL6J mice with Streptozotocin-induced diabetes	7.5 mg/kg b.w./day; 10 h prior to STZ	Insulin, glucose clearance, GLUT2 mRNA	Glucose, IL-16, TNF-α, pancreatic IL-6	N/A	Intraperitoneal	[176]
Wistar rats with Streptozotocin-induced diabetes	80 mg/kg b.w./day; 45 day	Insulin, SOD activity, CAT activity, GPx activity, glutathione activity	Kidney and liver: morphological changes, oxidative stress, TBARS, HP	30	Intraperitoneal	[177]
Swiss albino mice with Streptozotocin-induced diabetes	10 mM; 10 µL/mouse i.p.; 28 days and 106 BMCs, a single injection	Insulin, islet regeneration, SOD activity, catalase activity, GPx activity	Glucose, MDA levels	40	Intraperitoneal	[178]
Wistar rats with alloxan-induced diabetes	0.08 mg/kg b.w./day; 21 days	Hemoglobin, glutathione, GPx activity	Glucose, HbA1c, TBARS, SDH activity	36	Oral	[179]
Wistar rats with alloxan-induced diabetes	0.1 mg/kg b.w.; 2 h		Glucose	N/A	Oral	[180]

### 3.5. Kidney Diseases

Acute kidney disease (AKD) and chronic kidney disease (CKD) have led to several cases of mortality worldwide. An increase in inflammation and decreased antioxidant activity are mostly seen in kidney diseases and in hemodialysis patients. The supplementation of curcumin has shown favorable effects on renal diseases, mainly due to its anti-inflammatory and anti-oxidant properties. Curcumin has decreased renal damage and inflammation by reducing the expression of inflammatory cytokines such as IL-1β, IL-6, TNF-α, adiponectin (which is associated with arterial stiffness, leading to death) and cystatin in rats with adenine-induced CKD [181].

Nuclear factor-erythroid-2-related factor 2 (Nrf2) is a crucial transcription factor, and in the case of oxidative stress, Nrf2 translocates into the nucleus and induces the production of detoxifying enzymes. The upregulation of transcription factor Nrf2 was seen in the kidney upon the administration of curcumin; this upregulation led to an increase in glutathione reductase and thereby exhibited the antioxidant property by decreasing glutathione levels [182]. Diabetic nephropathy (DN) is the cause of end-stage renal failure, and inflammation plays an important part in the development and progression of DN. Curcumin prevents inflammation by renal macrophage infiltration and modulates transcription factors such as AP-1 and chemokines such as IL-1,IL-6. The oral supplementation of curcumin at 100 mg/kg/day for 8 weeks in STZ-induced diabetic rats prevented macrophage infiltration by inhibiting the activity of NF-κB, IκBα and regulated MCP-1 at the nuclear level, thereby preventing glomerular injury and damage [183]. Curcumin analogues in diabetic rats were administered at 5 mg/kg/day for 6 weeks, causing a similar reduction in kidney inflammation via the inhibition of the JNK pathway and diabetes-related histone acetylation [184]. Chemotherapeutic agents such as cisplatin cause acute kidney injury. Curcumin prevents the mitochondrial bioenergetics alterations and redox balance by preventing the increase in the mitochondrial fission protein and decreasing NAD± dependent deacetylase sirtuin-3 in acute kidney injuries [185]. Heavy metals cause nephrotoxicity due to ROS overproduction, decrease the endogenous antioxidant property and suppress the autophagy flux (leading to cell damage). Curcumin modulates autophagy via the modulation of Akt/mTOR and by increasing the adenosine monophosphate-activated protein kinase (AMPK) and extracellular signal-dependent kinase (ERK) pathways [186]. Curcumin administered orally in wistar rats at 400 mg/kg/day (with AKI via a dose of potassium dichromate) could preserve mitochondrial bioenergetics by increasing the expression of mitochondrial transcription factor A and bring peroxisome proliferator-activated receptor gamma coactivator 1-alpha (PGC-1α) back to a normal level [186]. Therefore, curcumin can be potentially used to treat renal diseases.

### 3.6. Antioxidant

Oxidative stress results from an imbalance between oxidants and antioxidative measures. It is hypothesized that damage from reactive oxygen species (ROS) and reactive nitrogen species (RNS) results in many chronic diseases (atherosclerosis, Alzheimer’s disease, liver disease) and the senescence of cells [187,188,189,190]. Curcumin has potent antioxidant properties due the fact that it has multiple functional groups including the β-diketo group, carbon–carbon double bonds and phenyl rings containing varying amounts of hydroxyl and methoxy substituents. These properties allow curcumin to protect lipid membranes from peroxidation induced by oxidation agents [191]. In fact, one study has shown that curcumin was more effective as an antioxidant than α-tocopherol [192].

Curcumin has multiple pathways to act as a direct antioxidant. Firstly, curcumin acts as an ROS (specifically H_2_O_2_) scavenger, as shown in vitro by Ak et al. [193]. Secondly, curcumin, through its phenolic or central methylenic groups, is associated with its hydrogen donor capacity [194]. Whatever et al. proved that the enol form of curcumin is more stable than the diketo form and that the bond-dissociation energy (BDE) of the phenolic O:H bond is lower than the BDE of the central O:H bond. Therefore, the hydrogen ion abstraction takes place in the phenolic form [195,196]. Thirdly, curcumin degradation products (ferulic acid and vanillin) under basic pH can act as potent antioxidants [191,197]. Lastly, curcumin can chelate heavy metal ions such as ferrous ions through its functional carbonyl group [193].

Additionally, curcumin exhibits indirect effects that combat oxidative stress on the cells. High-dose curcumin administration in albino rats by Faten et al. has shown the increased activity of antioxidant enzymes such as superoxide dismutase, catalase, glutathione peroxidase and glutathione-S-transferase (GST) in different tissues [198]. Furthermore, curcumin increased the mRNA expression (by 2–12 times) and protein levels (by 2–6 times) of antioxidant enzymes including glutamyl-cysteine ligase, quinone oxidoreductase and heme oxygenase 1 (OH-1) in human islet cells [199,200]. The expression of HO-1 was induced by curcumin through the activation of the Nrf2/antioxidant-responsive element (ARE) pathway in rat kidney epithelial cells [201]. Curcumin also increased the expression of the heat shock protein HSP70 [202]. Several studies have shown that curcumin inhibits phase 1 enzymes and activates phase 2 enzymes, leading to reduced toxic metabolites and increased antioxidants effects [203,204,205,206]. Curcumin acts indirectly to reduce oxidative stress through the inhibition of inflammatory pathways through the inhibition of NF-κB, which will be discussed later in the article.

Paradoxically, curcumin can selectively induce oxidative stress in cancer cells, leading to apoptosis and autophagy [207,208,209]. This was further proven when N-acetyl cysteine or glutathione was added and the curcumin effect was nulled [209,210]. The etiology of the paradoxical action of curcumin is unclear, but one study points to the significantly higher intake of curcumin in cancer cells [211]. Multiple studies are leveraging curcumin in the treatment of different types of cancers.

Among the various benefits of curcumin, the regulation of ER (Endoplasmic Reticulum) stress by using curcumin is an important strategy in treating several diseases such as cancer [212], diabetes [213], osteroporosis [214] and neurodegenerative diseases [215]. ER stress is caused due to the accumulation of unfolded or misfolded proteins, leading to a stress response called unfolded protein response (UPR). Curcumin can regulate ER stress by causing cell apoptosis or cell survival based on the type of cell being examined. In normal cells, curcumin scavenges ROS and decreases UPR, thereby suppressing ER stress and inhibiting apoptosis. In the case of inflammatory diseases, curcumin activates the MAPK pathway and increases the proteins involved in apoptosis such as transcription factor 6, the glucose-regulating protein and the C/EBP homologous protein CHOP. In diabetes, ER stress has been shown to trigger beta cells dysfunction or cell death [216]. Curcumin suppresses NF-κB activity and reduces caspase-12 and caspase-3 levels (usually increased due to ER stress) [217]. In murine myelomonocytic leukemia cells, curcumin induced apoptosis by the generation of ROS, the cytosolic release of Ca^2+^ and the inducing of DNA damage [218,219]. In human lung carcinoma A-549 cells, curcumin prevented cell proliferation by inducing G2/M-phase arrest and increased p53 and p21 levels, which are hallmarks of ER stress [220]. Curcumin caused apoptosis via the activation of CHOP in human leukemia HL-60 cells [221]. The exact cellular mechanism underlying the effect of ER stress on cell death or cell survival still needs more evidence due to its dualistic response.

### 3.7. Gut Microbiota

The exceptionally complicated and abundant microbial community inhabits the GI tract, with 100 trillion bacteria which are, remarkably, 10–100 times greater than the number of eukaryotic cells [222]. Furthermore, the gut environment differs markedly between different anatomical regions regarding physiology, substrate availability, digesta flow rates, host secretions, oxygen tension and pH [223,224]. Aside from the poor systemic bioavailability of curcumin, it is expected to be found at high concentrations in the gastrointestinal tract after oral administration. Thus, it is suspected that curcumin could exert direct regulative effects on the gut microbiota, which could explain the paradox between curcumin’s poor systemic bioavailability and its widely reported pharmacological effects (Table 4).

### 3.8. Inflammatory Bowel Disease (IBD)

TNF blockers, immunosuppressants and anti-inflammatory medications are commonly used to treat IBD, but due to the insufficient results and high cost involved in the treatment, there has been a need for alternatives. Bioactives have antioxidant and anti-inflammatory activity that could be used to effectively treat or prevent IBD. The use of curcumin in preclinical studies has suggested that it can target various molecular and cellular pathways involved in IBD pathogenesis. Recent studies have shown that the various molecular signaling pathways that participate in IBD development are targeted by curcumin, including PPAR-gamma, P13K, TLR-4, Akt, mTOR, ERK5, AP1, TGF-β, PAK1, Wnt, β-catenin, Shh, Rac1, p38MAPK, EBPα, NLRP3 inflammasome, Nrf2, Notch-1, AMPK, STAT3 and MyD-88 [230,231] Autophagy suppression has been linked with an excessive inflammatory response in IBD. In this regard, curcumin has shown an autophagy-regulating property by reducing the expression of genes such as Beclin-1, autophagy-related gene 5 and LC3II. In addition, curcumin has shown anti-apoptotic activity by inhibiting apoptotic cell death, thereby preventing damage to the intestinal epithelial barrier [232]. Studies have shown that curcumin suppresses NF-κB in chondrocytes by reducing the expression of cyclooxygenase, prostaglandin E-2 and inflammatory cytokines [233]. Curcumin can also interact with transient potential vanilloid receptor 1 in inflamed tissues to prevent intestinal inflammation [234]. Curcumin analogues such as non-electrophilic curcumin are known to suppress colitis in mice by inhibiting pro-inflammatory signals. Numerous clinical studies have linked inflammatory diseases to NLRP3. We agree with Karthikeyan et al. (2018) that curcumin can be a potential NLRP3 inflammasome suppressant by in vivo studies, and this could be a promising treatment for IBD [235].

However, due to its iron reduction property, curcumin should be carefully used for treatment since poor iron absorption is already seen in IBD patients. Therefore, monitoring the erythroid parameters is essential. Although curcumin alone or in combination with other drugs could be used for treatment by optimising the dosage, rigorous randomized controlled and long-term clinical trials should be conducted to establish the role of curcumin in the treatment of IBD.

## 4. Anti-Microbial

The antimicrobial activity of curcumin dates back to the old days when it was used as an insect repellent in the house [236]. Later, it was introduced as a potential suppressor of microbial activity in the cotton and wool industries [237]. Curcumin and other antimicrobial compounds have been key ingredients in ointments for skin protection and wound-dressing properties [238]. Several studies have reported the broad-spectrum antimicrobial activity for curcumin, including antibacterial, antiviral, antifungal and antimalarial activities.

### 4.1. Antiviral

Antiviral drugs are in high demand due to increasing viral infections globally and the lack of preventive and therapeutic options [238]. The first known antiviral activity of curcumin dates back to the 1990s, with the discovery that curcumin inhibits HIV viral protease in vitro. Since then, several studies have been conducted to understand its mechanism of action on different types of viruses. Each stage of the viral replication cycle, such as attachment/penetration, genome replication, gene expression, assembly and release, has been an attractive target for the effective inhibitory activity of curcumin. During the attachment step, the uptake of viral particles by binding to the receptors on the host cell membrane surface and entry into the host cell takes place by receptor-mediated endocytosis [239]. As a result, Curcumin has shown effective activity:

1. Against the viral envelope proteins by (a) modulating the membrane lipid bilayer of the host [240], (b) inhibiting its entry by interacting with viral surface proteins and reducing viral particle production [241], (c) disrupting the integrity of the viral membranes [242].
2. By targeting replication in two ways: (a) targeting the viral replication machinery and (b) modulating cellular factors to interrupt the replication process [243,244].

#### 4.1.1. Human Immunodeficiency Virus (HIV)

Curcumin has been shown to impact the HIV function at several stages of the virus lifecycle. Ferreira et al. conducted a study to understand the anti-inflammatory activity of curcumin in the female genital tract, which leads to the downregulation of tight junction (TJ) proteins, resulting in barrier loss and thereby allowing HIV-1 to traverse the genital epithelium and infect the host. The treatment of genital epithelial cells with 5 µM curcumin reduced the expression of virus replication marker p24 and protected the epithelial barrier by preventing TJ protein downregulation, thus reducing the HIV infection rate [245]. Curcumin can inhibit HIV replication by interacting with the viral integrase, protease and trans-activator of the transcription (Tat) protein. Docking studies have suggested that curcumin could bind effectively to the active site of HIV-1 protease [246]. Pretreatment with curcumin has inhibited the induction of proinflammatory cytokines such as Il-6, TNF and chemokines Il-8, IP-10, RANTES, eotaxin, MIP-1α (Macrophage Inflammatory Protein-1 Alpha) and MCP-1. In one study, curcumin degraded the Tat protein through a proteasomal pathway [247] and reduced its transactivation in HIV-1-infected cells. Even curcumin analogues, such as curcumin A (which lacks the β-diketone moiety of curcumin), have been tested against HIV-1 [248]. This study showed that curcumin A lowered late viral genome copy levels and could inhibit the early reverse transcription of the virus [248]. The therapeutic activity of curcumin is due to its ability to activate heme oxygenase-1, thereby inhibiting HIV-1 [249]. Curcumin-stabilized silver nanoparticles have shown promising activity by lowering HIV-LTR (Long Terminal Repeat) expression and lowering the expression of TNF-α, IL-6, IL-1β and NF-κB [250]. Collectively, these studies show curcumin’s potential against HIV-1.

#### 4.1.2. Severe Acute Respiratory Syndrome Coronavirus 2 (SARS-CoV-2)

Wen et al. (2007) have shown that curcumin can inhibit SARS-CoV-1 replication in the cultures of Vero E6 cells (EC_50_ of > 10 µM) [251]. Docking studies have concluded that curcumin could bind to target receptors such as protease, spike glycoprotein-receptor binding domain and PD-ACE2 (Angiotension Converting Enzyme-2). The ability of curcumin to modulate a wide range of molecular targets that are responsible for the attachment and internalization of SARS-CoV-2 could be used to effectively manage the coronavirus infection. Furthermore, Curcumin could block the entry of viruses into the cell by altering surface protein structures in the virus. Adding to this, a molecular docking study indicated that curcumin could bind to ACE2 to inhibit COVID19 entry into the cell. Curcumin could also interact with the viral protease, such as the main protease, which could be a potential therapeutic target [252]. Due to growing evidence on the effect of curcumin on interferons in different viral diseases, curcumin could trigger innate immunity by stimulating the production of interferon-stimulating genes and cytokines, as seen in the study on a porcine epidemic diarrhea virus model. Curcumin has played an important role in reducing the expression of crucial chemokines and cytokines such as IFN-γ, MCP-1, IL-6 and IL-10 in lung infection [253] and against the human RSV, preventing viral replication, which could be used to treat pulmonary inflammation due to COVID-19 infection. Reduction in the ACE2 expression could decrease the risk of renal damage. In this regard, curcumin could upregulate ACE2, leading to improved renal blood flow [254]. Curcumin can be used as an effective anti-fibrotic agent in kidneys [143]. To sum up, the antiviral and anti-inflammatory activity of curcumin can be helpful in both preventing and treating COVID-19. Further in vitro studies could help us better understand the mechanism of action, if any exists.

#### 4.1.3. Influenza A Virus (IAV)

Curcumin has been shown to inhibit NF-κB signaling, which is required for IAV replication. Curcumin or its analogues have been shown to inhibit IAV by preventing entry, inhibiting replication or preventing viral exit. A study by Dai et al. showed that curcumin interferes with early-stage virus gene expression and replication and inhibits several IAV-induced toll-like receptor signaling pathways including TLR2/4/7, MyD88, TRIF and TRAF6 [255,256]. Additionally, curcumin reduced IAV replication and lung injury in an in vivo animal model, which explains its role in combating infection and viral-induced disease [257]. Another study by Han et al. made a similar observation on mice infected with the IAV strain PR8 and fed 30 or 100 mg/kg of curcumin. Curcumin-treated mice had lower levels of MCP-1, IL-6 and TNF-α in bronchoalveolar lavage fluid and lung tissues as compared to untreated mice [257]. Curcumin analogues such as monoacetylcurcumin have inhibited plaque formation (IC_50_-0.2 µM). Although curcumin and MAC mildly reduce neuraminidase activity, they act via different mechanisms to inhibit IAV. The authors have suggested a combined use of the two for better activity [258]. A study by Lai et al. on MDCK cells treated with curcumin showed reduced mRNA levels of the IAV M gene in infected cells. Additionally, curcumin reduced lung pathology in in vivo treated mice [259].

#### 4.1.4. Herpes Simplex Virus (HSV)

Curcumin inhibited plaque formation by 88% and blocked viral adsorption by 92% in HSV1- and HSV2-infected Vero cell lines at a concentration of 30 μM. Curcumin treatment at a 5 μM concentration in primary human GECs reduced HSV-2 replication 1000-fold compared to the control group, and 50 μM of curcumin showed 100% inhibition [245]. To enhance the bioavailability of curcumin, it was encapsulated by Poly-(Lactic-Co-Glycolic Acid) and delivered via an intravaginal route against HSV-2 infection in mice. The results showed that curcumin-PLGA had no effect on the mice’s survival following the low or lethal dose of HSV-2 [260].

#### 4.1.5. Dengue Virus (DENV)

Curcumin reduced the plaque formation of all four strains (DENV-1–4) examined in LLC-MK2 cells, with limited toxicity (CC_50_ of 59.42 μM). Another study showed that curcumin inhibits DENV-2 by the indirect interaction with cellular systems rather than directly on viral function. A study by Balasubramanian et al. evaluated the anti-DENV (*Dengue virus*) properties of curcumin and other synthesized analogues. Curcumin and the analogues showed inhibitory activity on viral protease activity (IC_50_ 36–66 μM) [261]. The MOA of curcumin was through cellular lipid metabolism, as it downregulated acetyl-CoA carboxylase and fatty acid synthase and lowered the lipid droplet formation, which is usually seen in a DENV infection. Mainly, actin filament disorganization and defects in polymerization were seen after the curcumin treatment. Therefore, curcumin shows anti-DENV activity by actin filament organization, cell lipogenesis and viral enzymes [261,262].

#### 4.1.6. Enterovirus 71 (EV71)

Huang et al. evaluated the activity of curcumin against EV71 in HT29 human intestinal epithelial cells. A 10 μM concentration of curcumin reduced the protein expression during the early stage of infection and the genome replication of the virus and prevented EV-71-induced cell death. Usually, cells infected with EV71 show the phosphorylated residue Tyr311 of protein kinase C-delta [263], but it was reduced in curcumin-treated cells. Lin et al. evaluated curcumin-derived carbon quantum dot formulations (Cur-CQD) against EV71 [264]. This formulation increased the water solubility of curcumin due to a better antiviral activity. In addition, cur-CQD lowered the expression of viral proteins such as structural protein VP1 and non-structural proteins such as 3CD^pro^ and 3D^pol^ in a dose-dependent manner. The treatment also reduced the amount of viral mRNA and proteins that were detected in the brain and limb muscle tissue [252].

### 4.2. Antifungal Activity

Curcumin has the potential to be used as an antifungal against a wide range of fungi in in vitro and in vivo studies including cryptococcus, candida, trichophyton and Paracoccidioides [265,266]. With the emerging antifungal resistance, candida and other fungi species, there is a need for novel antifungal agents [267,268,269]. In addition, traditional anti-fungal medications such as azoles and polyenes possess serious side effects, most commonly resulting in kidney damage. On the other hand, curcumin has displayed minimal toxicity in a few reports, but no long-term trials have been conducted to assess its safety [270,271,272]

The exact mechanism of curcumin is unknown, but evidence by Sharma et al. showed that curcumin affects candida by increasing the production of reactive oxygen species (ROS) through altering membrane ATPase activity, interfering with ergosterol synthesis and inducing apoptosis as a result of reactive oxygen species accumulation [273,274,275]. This was proved further by including an antioxidant that attenuated curcumin’s effects on the fungus. The effect of curcumin on fungal cells also extends to the inactivation of specific genes that affect growth and drug metabolism. Curcumin targets global suppressor thymidine uptake 1 (TUP1) in candida, leading to its transcription and inhibiting hyphae development. Curcumin restored the sensitivity to fluconazole, which might be due to its effect on the active transporters (ABC and MDR) of the drug [273,276]. Curcumin has phytochemical properties when combined with photodynamic therapy and can be genotoxic to many fungi (candida, aspergillus and dermatophyte) since it can prevent the repair process of DNA damage [277,278,279].

A study by Martinez et al. measured the minimal inhibitory concentration (MIC) of Curcumin against 23 human pathogenic strains of fungi in vitro. Although Curcumin was more potent in many strains of Paracoccidioides brasiliensis than fluconazole, the strain MG05 growth was inhibited at an MIC of 0.5 mg/L of curcumin compared to 16 mg/L of fluconazole. Curcumin exhibits the potential to be administered through multiple routes, including intravenous, topical and oral routes depending on the offending agent site of infection. One study isolated the samples of candida from HIV patients with oropharyngeal candidiasis and exposed them to curcumin, which inhibited 90% of the yeast [280]. A study conducted on a vulvovaginal yeast infection model in rats benefited from 1.0% curcumin local cream application [281].

Owning to the phytochemical properties of curcumin. Many studies have examined the effect of curcumin with light on candida biofilm growth and dermatophytes infection. For example, Brasch et al. found that curcumin plus visible light inhibited the growth of different dermatophytes [278]. In addition, an experiment conducted by Dovigo et al. showed that candida growth and biofilm formation were inactivated using curcumin with photodynamic therapy [277].

Curcumin possesses the potential to be used as a monotherapy or in combination with azoles or polyenes. Sharma et al. proved that, when used in combination with Amphotericin b, curcumin showed a synergistic effect and a reduced side effects profile [273]. This can be leveraged in the future to reduce the dosage and, in turn, the side effects of current anti-fungal medications.

### 4.3. Antibacterial

Several antibiotics are available against specific bacteria. However, due to the extensive use of drugs, it is challenging to eliminate pathogens from the human body due to developed resistance. So, it is important to naturally get rid of bacterial infections. Curcumin, a known spice, shows antibacterial activities against most gram-positive and gram-negative bacteria [282]. Curcumin is known to be a relatively unstable molecule, with a particle size of 500–800 nm, impairing cellular uptake and resulting in low bioavailability [283,284]. A study found that curcumin kills several pathogenic gram-positive bacteria such as *Staphylococcus aureus*, *Staphylococcus epidermidis* and Enterococcus, which are the main causative agent of skin diseases, pneumonia, meningitis and urinary tract infections in human beings [282]. In addition, curcumin suppresses the adherence of *Streptococcus mutants* to human tooth surfaces and the extracellular matrix protein [285]. Curcumin possesses a synergistic effect with important antibiotics such as cefixime, vancomycin and tetracycline against *Staphylococcus aureus* (*S. aureus*) [286,287,288]. However, very few studies have demonstrated the mechanism of the antibacterial activity of curcumin, which seems to differ depending on the strain being studied. For instance, studies have shown that the antibacterial activity of curcumin against Bacillus subtilis occurs through the inhibition of bacterial cell proliferation by blocking the assembly dynamics of FtsZ in the Z ring [289]. In the case of *Pseudomonas aeruginosa* (*P. aeruginosa*) infection, curcumin was shown to have anti-infective activity by affecting virulence, quorum sensing and biofilm initiation [236].

Moreover, these mechanisms have not been confirmed in the case of other bacterial genera and, hence, could not be generalized for all bacteria. Therefore, a detailed study on the antibacterial mechanism of curcumin, including a large number of bacteria from different genera, is required. Furthermore, due to the increase of resistance in Gram-positive and Gram-negative bacteria, there is an urgent need to identify and assess alternative antimicrobials, including those from plant materials with low human cytotoxicity. Curcumin I showed no toxic effect on human health, even when taken at doses as high as 8 g per day [290].

## 5. Clinical Trials with Curcumin

Numerous clinical trials have been conducted with curcumin, appraising its therapeutic and pharmacological benefits across different patient populations. A summary of the concluded trials is depicted in Table 5, which will allow the reader to get a quick insight into the disease/patient population of interest. In this study, we have only considered registered and completed trials in the Clinical Trials registry. Of all the clinical trials, trial number NCT00927485 has studied the role of curcumin on intestinal adenomas for a significant duration of over 5 years, whereas others have done so for only a limited period of time. Therefore, the obtained results should be taken with a grain of salt. Case in point, in trial number NCT04012424, the trial was conducted for only a period of two days. This indicates that further trials extending over longer periods are required. Another shortcoming that is observed in most of the trials is the low number of participants. For example, in trial number NCT03568513, the effect of curcumin on IBS was only studied in 4 people out of the 50 that were expected to enroll.

Additionally, the trial has been conducted at a single center and does not provide enough information about the physiological and genetic makeup of the participants, which has been shown to affect the intestinal microbial milieu. Thus, the trial from this and similar trials require further investigation and validation. In addition to the above, most of the trials available in the database have not posted the obtained results; this makes it difficult to conclude if their primary outcome was achieved. The open-label study of the curcumin CS complex in schizophrenia (trial number NCT01875822) (Sl No.18) concluded in 2012; no results are available in the study page or in the literature. Although curcumin exhibits beneficial pharmacological effects in cell and animal models, the results are not very well replicated in human subjects. This has drawn considerable skepticism, and curcumin has been labelled as a pan-assay interference (PAINS) compound in the case of different screening tests such as fluorescence interference, the covalent labelling of proteins, redox reactivity, etc. It is to be noted that these tests are limited to in vitro studies; the real results obtained from human trials and case reports are more valid than any theoretical warning to prove its activity. Additionally, there has not been any experiment to prove that the biological activity of curcumin is due to its unique structure. A paper [291] suggested that curcumin is a “bimolecular sensitive fluorescent probe.” This does not necessarily have to be related to a fluorescence interfering property. We also know that any molecule with an ability to interact with various targets could bring numerous side effects. When it comes to several in vivo studies, curcumin proved to be safe even at a very high dose. A clinical trial in healthy volunteers consuming 500 to 12,000 mg of curcumin showed no toxicity, but a very low serum availability was detected in 2 of the 6 patients who received the highest dose (10,000 mg and 12,000 mg) [292]. This could possibly be due to the genetic modifiers of curcumin metabolism or even to the preparation method of commercially obtained curcumin. The activity of curcumin has also been related to its metabolites, which are more of an advantage, as this could possibly be used to treat diseases with multiple causes such as cancer and diabetes. A letter to the editor by Burgos-Moron et al. entitled “The dark side of curcumin” [293] suggests that the cytotoxicity of curcumin and its ability to intercalate into DNA have been nullified by an experiment conducted by Kurien et al., stating that the cytotoxicity was not due to curcumin but due to the solvent used for the dissolution of curcumin (i.e., ethanol) [294]. The best possible explanation for the ineffectiveness of curcumin in certain studies could be the very low bioavailability or moderate biological activity of curcumin. The non-replication of activity is related to curcumin’s low bioavailability due to the high hydrophobicity caused by the cyclic rings in its structure. Studies have shown that the combination with piperine enhances the serum concentration, the extent of absorption and the bioavailability of curcumin. However, few clinical trials have employed this strategy. In this regard, further investigation is required.

**Table 5:** Effect of curcumin on the completed clinical trials.

Sl No.	Clincal Trial Identifier	Trial Title	No. of Participants	Inclusion Criteria	Year of Completion	Primary Outcome	Clinical Trial No.	Follow-Up Period
1.	NCT03085680	Curcumin and Function in Older Adults	21	Aged above 65 years with a CRP level greater than 1.0 mg/dL	2020	To examine the effects of dietary supplementation with curcumin on changes in physical function, walking speed (400 m walk test) and grip strength	2	90 days
2.	NCT03211104	Comparison of Duration of Treatment Interruption with or without Curcumin During the Off Treatment Periods in Patients with Prostate Cancer Undergoing Intermittent Androgen Deprivation Therapy	107	Patients with localized prostate cancer or metastatic prostate cancer at the time of diagnosis who received intermittent androgen deprivation therapy (IAD)	2015	To determine whether the period from the first interruption of the androgen deprivation therapy to the time when androgen deprivation therapy needs to be retreated differs between the curcumin group and placebo group	NA	180 days
3.	NCT04012424	The Effect of Premedication with Curcumin on Post-endodontic Pain	44	Patients in the age range of 20–55 years with acute pulpitis	2020	Change in postoperative pain after a single endodontic visit	N/A	2 days
4.	NCT04870060	Ability of Curcumin to Decrease Cytokines Involved in Mucositis in the Autologous Transplant	40	Patients aged 18 years and above with a creatinine clearance greater than 50 mL/min and a serum bilirubin level greater than 2 mg/dl	2015	To calculate TNFa, IL-1, IL-6, IL-8, IL-17, TGF-B, IFN-gamma and E2 levels	2	28 days
5.	NCT01543386	Effects of Curcumin on Vascular Reactivity	21	50- to 70-year-old smokers	2012	Changes in brachial flow-mediated dilatation	2	5 days
6.	NCT03568513	Effect of Curcumin on Gut Microbiota in IBS	4	Patients aged 10 to 18 years with diarrhoea-predominant IBS	2020	Alterations in gut microbiota	N/A	56 days
7	NCT03864783	The Effect of Curcumin on Liver Fat Content in Obese Subjects	39	BMI and haemoglobin greater than 30.0 kg/m^2^ and 7.5 mmol/L, respectively	2020	Curcumin’s effect on steatosis	N/A	42 days
8.	NCT04044417	Curcumin-Simvastatin-EDTA in the Treatment of Periodontitis	30	Patients aged 25 to 50 years suffering from at least a single posterior 2–3 wall periodontal pocket of depth	2018	Reduction in probing depth	4	180 days
9.	NCT04032132	Curcumin Paste as an Adjunctive Therapy in Periodontitis	24	Patients aged 25 to 45 years with at least a single posterior 2–3 wall periodontal defect of pocket depth	2018	Evaluate the influence of curcumin paste on the clinical outcomes of the surgical treatment	4	180 days
10.	NCT03746158	Interindividual Variation in Excretion of Curcumin	8	18–30-year-old healthy adults.	2019	Determine the concentration of curcumin and its metabolites in human fecal samples	N/A	28 days
11.	NCT01179256	Effect of Supplemental Oral Curcumin in Patients with Atopic Asthma	16	Patients aged 18–60 years on low- or medium-dose inhaled corticosteroids	2010	Improvement in post-bronchodilator FEV1	N/A	N/A
12.	NCT01246973	Oral Curcumin for Radiation Dermatitis in Breast Cancer Patients	686	Females aged 21–120 years	2015	To measure the Mean Radiation Dermatitis Severity Score	2	42 days
13.	NCT04119752	Effect of Curcumin on Microvascular Response and Tissue Oxygenation in Older People	28	Aged 60– 85 years with two or more risk factors for cardiovascular disease	2020	Changes in microvascular reactivity and tissue oxygen saturation.	N/A	120 min
14.	NCT02255370	Curcumin Associated with Thiopurin in the Prevention of Post-op Recurrence in Crohn Disease (POPCUR)	61	Patients aged 18 years and older with Crohn’s disease	2018	Rutgeerts endoscopic score	3	180 days
15.	NCT02298985	Curcumin Addition to Antipsychotic Treatment in Chronic Schizophrenia Patients	38	Patients aged 18–60 years with schizophrenia and a SANS greater than 30 points	2017	Positive and Negative Symptoms Scale (PANSS)	4	180 days
16.	NCT01383161	18-Month Study of Memory Effects of Curcumin	46	Aged 50–90 years with a modified Ischemic score of less than 4	2017	Change from the baseline to 18 months on the Brief Visual Memory Test-Revised	2	540 days
17.	NCT01333917	Curcumin Biomarkers	40	Healthy volunteers aged 40–80 years	2013	To understand the changes in gene expression, the ribonucleic acid (RNA) level and apoptosis	1	30 days
18.	NCT01875822	Open-label Study of Curcumin C-3 Complex in Schizophrenia	17	Patients aged 18–65 years with DSMIV schizophrenia and a SANS greater than 30	2012	To understand the change from the baseline negative symptoms: alogia, anhedonia, social withdrawal and lack of motivation	2	112 days
19.	NCT02978339	A Study Evaluating the Safety and Efficacy of Curcumin in Patients with Primary Sclerosing Cholangitis (PSC)	15	Diagnosed with primary sclerosing cholangitis with alkaline phosphatase >1.5×	2019	Change in Serum Alkaline Phosphatase (SAP)	2	84 days
20.	NCT04208334	The Effect of Curcumin for Treatment of Cancer Anorexia-Cachexia Syndrome in Patients with Stage III-IV of Head and Neck Cancer (CurChexia)	20	Patients with stage 3–4 head and neck cancer	2021	To measure muscle mass	2	60 days
21.	NCT01925287	Oral Bioavailability of Curcumin from Micronized Powder and Liquid Micelles in Healthy Young Women and Men	23	Healthy volunteers with a normal range blood chemistry value	2013	To determine total curcumin, demethoxycurcumin and bisdemethoxycurcumin after deconjugation with beta-glucuronidase	1	24 h
22.	NCT02104752	Curcumin as a Novel Treatment to Improve Cognitive Dysfunction in Schizophrenia	39	Volunteers diagnosed with DSM-5 schizophrenia with a corrected vision of at least 20/30	2017	Measurement and treatment research to improve cognition in schizophrenia	1	56 days
23.	NCT02369549	Micro-Particle Curcumin for the Treatment of Chronic Kidney Disease	518	Patients with an eGFR between 15 and 60 mL/min/1.73 m^2^ with a minimum of 300 mg of protein in urine or with a albumin/creatinine ratio of at least 300 mg	2020	Change in albuminuria and the Estimated Glomerular Filtration Rate (eGFR)	3	180 days
24.	NCT02439385	Avastin/FOLFIRI in Combination with Curcumin in Colorectal Cancer Patients with Unresectable Metastasis	50	Colon or rectal cancer patients aged above 19 years with an ASA score of less than 3	2019	To evaluate progression-free survival in colorectal cancer patients	2	730 days
25.	NCT02474953	A Study to Compare the Pharmacokinetic Profile of a Proprietary Curcumin Formulation to a Comparator Curcumin Product (15PCHB)	12	Volunteers aged 18–45 years with a BMI that is 18–29.9 kg/m^2^(±1 kg/m^2^)	2015	To measure the maximum concentration of curcumin and time until the max concentration of curcumin	1	48 h
26.	NCT04421716	Testing the Bioavailability of Phytonutrients, Curcumin and Ursolic Acid	18	Men aged 18 years or older	2021	To evaluate the number, frequency, duration and relation of toxicity events to CURC and UA, the peak serum concentration, the half-life and the time taken to reach the maximum concentration	1	14 days
27.	NCT04258501	Exploratory Study of Efficacy on Selected Natural Extracts Reducing Post Prandial Blood Glucose Response	72	20–50-year-old healthy individuals with a normal BMI	2012	Change in post-prandial blood glucose	NA	2 h
28.	NCT01035580	Trial on Safety and Pharmacokinetics of Intravaginal Curcumin	13	Volunteers aged 18–45 years currently using a birth control method	2012	To reach the maximum selected dose or maximum tolerated dose of intravaginal curcumin without a dose-limiting toxicity	1	14 days
29.	NCT01403545	Evaluation of Liposomal Curcumin in Healthy Volunteers	50	Volunteers in the age group of 18–45 years with a BMI between 18–27 kg/m^2^	2012	Safety and tolerability of increasing doses of intravenous liposomal curcumin	1	7 days
30.	NCT01225094	Curcumin to Prevent Complications After Elective Abdominal Aortic Aneurysm (AAA) Repair	606	Volunteers aged 18 years or above who have undergone the repair of AAA	2016	To measure urine IL-18, NT-ProBNP, hsCRP and serum creatinine	2	N/A
31.	NCT01160302	Curcumin Biomarker Trial in Head and Neck Cancer	33	Volunteers aged between 18–90 years willing to undergo tumor biopsies	2016	Change in tissue biomarkers and pharmacokinetics of microgranular curcumin	1	28 days
32.	NCT01917890	Radiosensitizing and Radioprotective Effects of Curcumin in Prostate Cancer	40	Aged between 50–80 years with relapsed or treated basal skin cancer and no severe hypertension	2013	Biochemical or clinical progression-free survival	N/A	365 days
33.	NCT00895167	The Effects of Oral Curcumin on Heme Oxygenase-1 (HO-1) in Healthy Male Subjects (CUMAHS)	12	Aged between 18–45 years with a BMI between 18 and 28 kg/m^2^	2009	The maximal HO-1 mRNA expression and HO-1 protein level in PBMCs	1	48 h
34.	NCT03542240	Effects of Curcumin Supplementation on Gut Barrier Function in Patients with Metabolic Syndrome	15	Waist Circumference—Female: ≥ 88 cm, Male: ≥ 102 cm B. Blood Pressure: ≥ 130/85 mm/Hg. Impaired fasting glucose or HbA1c fasting glucose ≥ 100 mg/dL or HgA1c ≥ 5.7 D. HDL-C—Females: < 50 mg/dL, Males: < 40 mg/dL E. Triglycerides ≥ 150 mg/dL	2020	Change in intestinal permeability and intestinal barrier function	N/A	365 days
35.	NCT00927485	Use of Curcumin for Treatment of Intestinal Adenomas in Familial Adenomatous Polyposis (FAP)	44	21–85 years with FAP (with an intact colon or who have had surgery)	2016	To determine the number of polyps and the size of polyps		5 years
36.	NCT01042938	Curcumin for the Prevention of Radiation-induced Dermatitis in Breast Cancer Patients	35	Females aged 21 years or above with a diagnosis of non-inflammatory breast adenocarcinoma	2011	Severity of dermatitis in the radiation treatment site in breast cancer patients	2	49 days
37.	NCT01490996	Combining Curcumin with FOLFOX Chemotherapy in Patients with Inoperable Colorectal Cancer (CUFOX)	41	18 years or above, diagnosed with metastatic colorectal cancer and with an ECOG status of 0 or 1	2017	Completion of dose escalation over two cycles of therapy	2	365 days
38.	NCT01975363	Pilot Study of Curcumin for Women with Obesity and High Risk for Breast Cancer	29	Females with an increased risk of breast cancer and a BMI between 25–40	2016	Determine the adherence, tolerability and safety of two doses of nanoemulsion curcumin	N/A	90 days
39.	NCT01859858	Effect of Curcumin on Dose Limiting Toxicity and Pharmacokinetics of Irinotecan in Patients with Solid Tumors	23	Aged above 19 years with adequate bone marrow, renal and hepatic function and an ECOG status of 0 or 1	2016	Maximum tolerated dose, pharmacokinetics of irinotecan and SN-38	1	28 days
40.	NCT04103788	Evaluation of Increased Absorption of a Curcumin Emulsion (CurQ+) in Healthy Volunteers	10	Aged between 21 and 75 years	2018	Comparative effect of differing serum sample preparation methodologies on curcumin absorption levels	N/A	6 h
41.	NCT01925547	Micellar Curcumin and Metabolic Syndrome Biomarkers	42	Total cholesterol > 5.2 mmol/L, LDL cholesterol > 3.4 mmol/L, Triglyceride > 2.26 mmol/L, CRP > 2 mg/L	2014	To measure the serum CRP level	2	42 days
42.	NCT01330810	Curcumin Pharmacokinetics	12	Aged between 16 and 65 years with a BMI in the range of 18–30 kg/m^2^	2012	To measure the AUC, C_max_, T_max,_ Ke, T_1/2_, V_d_ and bioequivalence of tissue curcumin concentration	1	48 h
43.	NCT02908152	Curcumin Supplement in Nonalcoholic Fatty Liver Patients	50	Patients diagnosed with type 2 diabetes with a CAP score greater than 263	2017	To measure hepatic steatosis	2	72 days
44.	NCT01201694	Phase I Study of Surface-Controlled Water Soluble Curcumin (THERACURMIN CR-011L)	28	Patients aged 13 or older with an ECOG status of 3 or better and normal organ and marrow function	2014	To measure the Maximum Tolerated Dose (MTD) of surface-controlled water-dispersible curcumin	1	28 days
45.	NCT04028739	Theracurmin vs. Curcumin Bioavailability Study	24	Healthy adults aged 19–60 years with a BMI of 18–30 kg/m^2^	2019	To compare the bioavailability of curcumin in healthy adults	NA	12 h
46.	NCT03795792	Oral Curcumin Administration to Remit Metabolic Syndrome	105	Men and women aged 20–55 years old with metabolic syndrome according to the ATP III criteria	2019	Remission of metabolic syndrome (≤2 components according to the ATP III criteria)	NA	3 months
47.	NCT00528151	A Randomized, Double-blind, Placebo-controlled Trial of Curcumin in Leber’s Hereditary Optic Neuropathy (LHON)	70	Aged 8 years or older with Leber’s hereditary optic neuropathy	2007	Visual outcome	3	1 year
48	NCT00889161	Curcumin in Pediatric Inflammatory Bowel Disease	11	8–18-year-old patients with IBD who have been on IBD medication for 3 months	2010	To determine the tolerability of curcumin in pediatric patients with inflammatory bowel disease	1	9 weeks
49	NCT01514266	Effect of Curcumin on Lung Inflammation	57	≥45-year-old patients with COPD and a stable clinical course	2010	Change in sputum dysplasia	NA	3 months
50	NCT00779493	Curcumin (Tumeric) in the Treatment of Irritable Bowel Syndrome: A Randomized-Controlled Trial (CuTIBS)	17	≥18-year-old patients who conform to the Rome III criteria	2009	The primary outcome will be defined as at least a 50% reduction in the irritable bowel severity score (IBSS)	4	6 months
51	NCT03329781	Modulation of Endotoxaemia Via Curcumin Intake in Healthy Overweight Adults (ENDOCUR)	16	18–45-year-old healthy individuals with a BMI ≥ 25 kg/m^2^	2018	Level of endotoxin in plasma	NA	21 days
52	NCT00094445	Trial of Curcumin in Advanced Pancreatic Cancer	50	≥45-year--old patients with unresectable adenocarcinoma of the pancreas	2014	6-month participant survival	2	6 months
53	NCT01750359	Efficacy and Safety Curcumin in Depression	40	20–60-year-old patients with a major depressive disorder	2011	Change in Hamilton Depression Rating Scale and Montgomery–Asberg Depression Rating Scale	4	6 weeks
54	NCT00181662	Pharmacokinetics of Curcumin in Healthy Volunteers	6	≥45year-old healthy female individuals	2007	Curcumin pharmacology	NA	NA
55	NCT03598205	Curcumin and Intravitreal Dexamethasone in Diabetic Macular Edema (DIABEC)	72	18–90-year-old patients with significant diabetic macular edema and a central retinal thickness of >300 microns	2019	Mean difference in central retinal thickness from baseline to 6 months	NA	6 months
56	NCT00641147	Curcumin in Treating Patients with Familial Adenomatous Polyposis	44	18–85-year-old patients with familial adenomatous polyposis	2016	The average number of polyps in the placebo arm at the end of the study is compared to the average in the curcumin arm	2	12 months
57	NCT04385979	Curcumin and Nanocurcumin in Oral Aphthous Ulcer	48	Patients with minor and recurrent aphthous ulcers with 48 h	2020	Wound size and pain score	NA	1 week
58	NCT01320436	Curcumin + aminosalicylic Acid (5ASA) Versus 5ASA Alone in the Treatment of Mild to Moderate Ulcerative Colitis	50	18–70-year-old patients with confirmed diagnosis of ulcerative colitis on a stable dose of ulcerative colitis medication	2014	The percentage of patients who achieve clinical remission compared between the two study arms	3	4 weeks
59	NCT03072992	“Curcumin” in Combination with Chemotherapy in Advanced Breast Cancer	150	18–75-year-old female patients diagnosed with breast carcinoma and adequate organ function	2019	Objective response rate, assessed with the Modified Response Evaluation Criteria in Solid Tumours (RECIST)	2	24 weeks
60	NCT00113841	Curcumin (Diferuloylmethane Derivative) With or Without Bioperine in Patients with Multiple Myeloma	42	Patients with multiple myeloma and adequate organ function	2009	Percent change of NF-kB protein expression in peripheral blood mononuclear cells	NA	4 weeks
61	NCT01909037	Exploratory non comparative Study to Evaluate the Efficacy of Highly Bioavailable Curcumin (Flexofytol) in Patients with Knee Osteoarthritis	22	45–80-year-old patients with osteoarthritis and a symptomatic knee for more than 6 months who can avoid using analgesics during the study	2012	Change in the serum levels of biomarkers of cartilage metabolism and inflammation	1	84 days
62	NCT00365209	Phase II A Trial of Curcumin Among Patients with Prevalent Subclinical Neoplastic Lesions (Aberrant Crypt Foci)	44	≥40-year-old patients with a >3 pack-year smoking history	2011	Change in prostaglandin E2 (PGE2) values found in rectal aberrant crypt foci (ACF) tissue	2	30 days
63	NCT02494141	Curcumin Therapy to Treat Vascular Dysfunction in Children and Young Adults With ADPKD	68	6–25-year-old patients with an ADPKD diagnosis and normal renal function	2021	Change in brachial artery flow-mediated dilation (FMD-BA) and aortic pulse-wave velocity (aPWV)	4	12 months
64	NCT04378972	Anti-inflammatory Effect of Curcumin, Homotaurine, Vitamin D3 on Human Vitreous in Patients with Diabetic Retinopathy	25	≥18-year-old patients with diabetic retinopathy requiring vitrectomy	2019	Analyze human vitreous samples’ pro-inflammatory cytokines	NA	7 days
65	NCT04972045	Bioavailability of Curcumin Capsules in Healthy Adult Subjects	12	18–55-year-old healthy subjects with a BMI of 18–28 kg/m^2^	2021	Measure Peak Plasma Concentration, area under the curve, Tmax and bioavailability	1	3 days
66	NCT01489592	Effect of Curcumin on Iron Metabolism in Healthy Volunteer (CURHEP)	18	18–35-year-old healthy adults with a BMI of 18–25 and no HFE mutation	2012	Maximal variation of the serum hepcidin level after the oral administration of curcumin	2	48 h
67	NCT01964846	Effect of Antioxidant Intake on Cardiovascular Risk	22	45–70-year-old healthy patients with a stable weight	2015	Change in the blood levels of anti- and pro-inflammatory markers	NA	2 weeks
68	NCT02100423	Curcumin and Cholecalciferol in Treating Patients with Previously Untreated Stage 0-II Chronic Lymphocytic Leukemia or Small Lymphocytic Lymphoma	35	≥18-year-old patients with a CLL or SLL diagnosis and adequate organ function	2018	Overall response rate (biologic response rate + complete response [CR] + partial response [PR]) based on NCI-WG (for CLL) and the Cheson criteria (for SLL	2	2 years
69	NCT03530436	Comparison of Curcumin Bioavailability	12	18–35-year-old healthy individuals	2018	Pharmacokinetics of curcuminoids (curcumin, demethoxycurcumin, bisdemethoxycurcumin) at different time frames	NA	24 h
70	NCT02529982	Curcumin Supplementation and Patients with Type 2 Diabetes	44	44–65-year-old patients with type 2 Diabetes Mellitus with a BMI of 18.5–30 kg/m^2^	2016	Fasting blood sugar, insulin, HbA1c, homeostatic model assessment of insulin resistance and change in pancreatic B-cell function	NA	10 weeks
71	NCT03066791	Turmeric and Curcumin on Sebum Production	30	18–50-year-old healthy individuals	2017	Sebum production	NA	8 weeks
72	NCT01514370	Dietary Supplement of Curcumin in Subjects with Active Relapsing Multiple Sclerosis Treated With Subcutaneous Interferon Beta 1a (CONTAIN)	80	18–60-year-old patients with multiple sclerosis under the treatment of IFN beta-1a for 6–12 months	2016	Number of subjects with active (new or enlarging) T2 lesions, as assessed by magnetic resonance imaging (MRI) at Month 12	2	24 months
73	NCT00475683	Curcumin for Prevention of Oral Mucositis in Children Chemotherapy	8	5–30-year-old patients diagnosed with cancer who received doxorubicin containing chemotherapy	2010	Measured change of an objective measurement of oral mucositis	3	6 weeks
74	NCT00164749	A Pilot Study of Curcumin and Ginkgo for Treating Alzheimer’s Disease	36	≥50-year-old patients of Chinese ethnicity with a progressive decline in memory ≥6 months	2006	Measured change in the isoprostane level in plasma and the A-beta level in serum	2	6 months
75	NCT02152475	Photodynamic Therapy (PDT) for Oral Disinfection	30	20–35-year-old healthy adults who do not perform any oral hygiene	2013	Microbiological analysis by the total number of colony-forming units	1	2 h
76	NCT01831193	Effect of Oral Supplementation with Curcumin (Turmeric) in Patients with Proteinuric Chronic Kidney Disease	120	18–70-year-old patients diagnosed with proteinuric chronic kidney disease taking ARB or ACEi	2014	Change in proteinuria	3	8 weeks
77	NCT02556632	Prophylactic Topical Agents in Reducing Radiation-Induced Dermatitis in Patients with Non-inflammatory Breast Cancer (Curcumin-II)	191	≥21-year-old patients diagnosed with non-inflammatory breast cancer or carcinoma in situ who are undergoing radiation therapy	2016	Measured mean Radiation Dermatitis Severity (RDS) score, incidence of moist sesquamation and change in the severity of skin reactions using RDS	2	1 week post-radiation chemotherapy
78	NCT04465851	Effect of Ferrous iROn and cUrcumin sTatus on Inflammatory and Neurotrophic markErs (Fe-ROUTINE)	155	18–40-year-old healthy individuals	2020	To assess the influence of curcumin administration on ferrous iron supplementation-associated inflammation	NA	42 days
79	NCT00192842	Gemcitabine With Curcumin for Pancreatic Cancer	17	≥18-year-old patients suffering from advanced or metastatic pancreatic adenocarcinoma with no prior therapy	2010	time to tumor progression	2	NA
80	NCT00099710	Curcumin in Patients with Mild to Moderate Alzheimer’s Disease	33	≥50-year-olds with a diagnosis of Alzheimer’s disease	2007	Measured safety and tolerability of curcumin	2	12 months
81	NCT01712542	Curcumin Bioavailability in Glioblastoma Patients	15	≥18-year-old patients with glioblastoma	2013	Measured concentration of curcumin in glioblastoma	NA	At time of tumor resection
82	NCT01022632	Effect of Curcumin as Nutraceutical in Patients of Depression	60	18–65-year-old patients with a diagnosis of depression	2010	Measured response and mean change in the Hamilton Depression Rating Scale (HAM-D17)	NA	6 weeks
83	NCT03144882	Evaluation of Curcumin’s Effect on Inflammation in Hemodialysis Patients	71	≥18-year-old clinically stable patients receiving hemodialysis	2017	Measured mean Interleukin-6 levels	NA	1 year
84	NCT03141918	Effect of Supplementation of Bioactive Compounds on the Energy Metabolism of People Living With HIV/AIDS	20	18–70-year-old patients with HIV receiving antiretroviral therapy ≥6 months	2017	Measuring the oxidation of energetic substrates; evaluation at rest	NA	10 days
85	NCT01740323	Phase II Study of Curcumin vs. Placebo for Chemotherapy-Treated Breast Cancer Patients Undergoing Radiotherapy	30	≥18-year-old female patients undergoing breast radiotherapy	2018	Measured change in NF-kB DNA binding, Plasma TNF-alpha, sTNFR2, IL-1ra, IL-6 and CRP	2	6 weeks after the completion of radiotherapy
86	NCT04107987	Berberine, Curcumin, Inositol, Banaba and Chromium Picolinate in Patients with Fasting Dysglycemia	148	18–75-year-old patients with impaired fasting glucose who are not on treatment	2019	Measured progression of dysglycemia	3	3 months
87	NCT00027495	Curcumin for the Prevention of Colon Cancer	NA	≥18-year-old healthy individuals	2007	To determine the pharmacokinetics and measure the Maximum Tolerated Dose (MTD)	1	72 h
88	NCT04723849	Efficacy Evaluation of a Mixed Compound of Antioxidants in Terms of Endothelium Damage/Function in Pediatric Subjects with Obesity. (OBELIX)	48	6–17-year-old patients with a BMI > 95% for their age based on the CDC standard	2020	To test the effects of a mixed compound including curcumin on endothelium in a cohort of pediatric subjects with obesity	NA	6 months
89	NCT00768118	A Nutritional Supplement Capsule Containing Curcumin, Green Tea Extract, Polygonum Cuspidatum Extract, and Soybean Extract in Healthy Participants	11	≥18-year-old healthy individuals	2008	Measure the magnitude of change in the blood lymphocyte NF-kB level	NA	15 days
90	NCT02017353	Effect of Curcumin Addition to Standard Treatment on Tumour-induced Inflammation in Endometrial Carcinoma	7	≥18-year-old female patients with endometrial carcinoma and no life-threatening metastases	2016	Measured change in the inflammatory markers in peripheral blood from the baseline	2	21 days
91	NCT00792818	The Efficacy and Safety of Curcuma Domestica Extracts and Ibuprofen in Knee Osteoarthritis	367	50–75-year-old patients diagnosed with primary osteoarthritis	2012	Measured change in mean Western Ontario and McMaster Universities Osteoarthritis (WOMAC) pain subscale	3	12 months
92	NCT03290417	Correlative Analysis of the Genomics of Vitamin D and Omega-3 Fatty Acid Intake in Prostate Cancer	37	Patients diagnosed with prostate cancer who are on active surveillance	2019	Measured gene expression of very low and low-risk prostate cancer patients on active surveillance	NA	12 months
93	NCT00525421	A Clinical Study of Curcuminoids in the Treatment of Oral Lichen Planus	20	≥21-year-old patients diagnosed with lichen planus	2009	Measured percent change from the baseline to two weeks in the symptoms and signs of oral lichen planus	2	2 weeks
94	NCT02337192	Antimicrobial Photodynamic Therapy Applied in Orthodontic Patients.	24	18–50-year-old healthy individuals with fixed orthodontic treatment	2014	Microbiological analysis by the total number of colony-forming units (CFU)	1	1 h
95	NCT01288859	Physiological Effects of New Polyphenol-enriched Foods in Humans	10	18–45-year-old healthy individuals	2011	Measured serum polyphenol concentrations, urinary excretion of total polyphenols and the number of total fecal polyphenols	NA	24 h
96	NCT01029327	Effects of Curcumin on Postprandial Blood Glucose, and Insulin in Healthy Subjects	15	≥18-year-old healthy individuals	2009	To study the effect of curcumin on the postprandial blood glucose and plasma concentrations of insulin	NA	NA
97	NCT02815475	Turmeric Anti-Inflammatory and Cell-Damage Trial (TACT)	90	18–80-year-old healthy individuals	2016	Measured change from baseline DNA methylation analyses and baseline oxidative stress determination	NA	6 weeks
98	NCT03769857	NEM^®^ + BIOCURC^®^ Versus Placebo in Exercise-induced Joint Pain, Stiffness, & Cartilage Turnover in Healthy Men & Women	84	40–75-year-old healthy adults with no diagnosis of joint arthritis	2019	Measured exercise-induced cartilage turnover	NA	2 weeks
99	NCT03621865	A Comparative Pharmacokinetic Study to Evaluate the Ability of a New Formulation to Enhance Curcuminoids Bioavailability (TURBIO)	30	18–45-year-old healthy individuals with a normal BMI and a stable weight	2018	Measured dose-normalized AUC of total curcuminoids plasmatic concentration	NA	24 h
100	NCT03289832	Effect of Orally Delivered Phytochemicals on Aging and Inflammation in the Skin	25	18–70-year-old healthy individuals willing to avoid sun exposure and follow a diet	2019	Measured change in erythema 1, 2 and 3 Days after UV exposure	NA	10 days
101	NCT03140657	The Effects of Nanocurcumin on Treg Cells and Th17 Cells Responses in Ankylosing Spondylitis Patients	24	23–46-year-old patients with a diagnosis of ankylosing spondylitis	2018	Assessments of ankylosing spondylitis signs and symptoms (BASDI)	2	4 months
102	NCT03192059	Study of Pembrolizumab, Radiation and Immune Modulatory Cocktail in Cervical/Uterine Cancer (PRIMMO)	43	≥18-year-old female patients with endometrial, cervical or uterine malignancy refractory to treatment	2021	Measured efficacy (objective response rate) at week 26 according to the immune-related response criteria (irRC)	2	156 weeks
103	NCT03530787	Cosmetic Effects of Topical Acetyl Zingerone	31	30–60-year-old healthy individuals	2018	Measured change in wrinkle appearance and skin pigmentation	NA	8 weeks
104	NCT03493997	Multicentre International STudy for the Prevention with Ialuril^®^ of Radio-induced Cystitis (MISTIC)	100	≥18-year-old male patients who planned to receive primary therapy for prostate cancer	2018	Measured rate of patients who stopped treatment with intravesical or oral ialuril due to intolerance or adverse events	2	12 months
105	NCT04849182	Vertistop^®^ D and Vertistop^®^ L in Preventing Recurrence of High-recurrence BPPV	128	18–85-year-old patients with benign paroxysmal positional vertigo (BPPV)	2020	Measured number of BPPV recurrences in patients supplemented with Vertistop D	NA	6 months
106	NCT02099890	The Effect of Diet on Chronic Inflammation and Related Disorders Following Spinal Cord Injury	20	≥18-year-old patients with a spinal cord injury	2015	Measured change from the baseline in the nerve conduction velocity of somatic nerves at 3 and 6 months	3	6 months
107	NCT03483376	aPDT for the Remediation of Dental Black Stain	30	≥12-year-old patients with a dental black stain in at least two teeth	2020	Area and depth of color of the black stain	NA	6 months
108	NCT00235625	Curcuminoids for the Treatment of Chronic Psoriasis Vulgaris	12	18–75-year-old patients with chronic plaque-type psoriasis	2007	Physicians Global Assessment (PGA) of change	2	16 weeks
109	NCT04382040	A Phase II, Controlled Clinical Study Designed to Evaluate the Effect of ArtemiC in Patients Diagnosed With COVID-19	50	≥18-year-old patients with a diagnosis of SARS-CoV-2 infection who are hospitalized and are in stable condition	2020	Time to clinical improvement, defined as a national Early Warning Score 2 (NEWS2) of ≤ 2, maintained for 24 h, and measurement of adverse events	2	2 weeks
110	NCT03150966	The Immunomodulatory Effects of Oral Nanocurcumin in Multiple Sclerosis Patients	41	18–65-year-old patients who are diagnosed with multiple sclerosis	2017	Measurement of the Expanded Disability Status Scale (EDSS)	2	6 months
111	NCT02442453	Effect of Scaling and Root Planing Along with Topical Application of Commercially Available Curcuma Longa Gel on Superoxide Dismutase and Malondialdehyde Levels in Saliva of Chronic Periodontitis Patients	100	30–55-year-old healthy individuals with chronic generalized periodontitis	2014	Measurement of the superoxide dismutase antioxidant enzyme levels in the saliva of chronic periodontitis subjects	4	1 month
112	NCT02909621	Evaluation of FLEXOFYTOL^®^ Versus PLACEBO (COPRA)	150	45–80-year-old patients with knee osteoarthritis	2017	Measuring the variation in the serum levels of the sColl2-1 biomarker between T0 and T3 by specific immunoassays and the variation in the global assessment of disease activity by the patient using a visual analogue scale (VAS)	NA	6 months
113	NCT04439981	Curcuma Extract Beneficial for Muscle Damage	20	14–18-year-old healthy male individuals	2019	Change in lactic acid, Hb, IL-6 and creatinine kinase	NA	21 days
114	NCT02251678	Evaluate the Effect of Elimune Capsules	21	≥18-year-old patients with plaque psoriasis with or without arthritis	2015	Individual subject serum levels of biomarkers (CRP, TNFa, IL-6, IL-12)	1	28 days
115	NCT04633551	Vascular Inflammation and Anti-inflammatory Supplements After Adverse Pregnancy Outcomes (VIA)	8	18–45-year-old female patients who had a singleton pregnancy of < 3 years complicated by an adverse pregnancy outcome (APO)	2021	Measurement of blood pressure, arterial stiffness, augmentation index and endothelial function	NA	1 month
116	NCT02834078	Effect of BGG on Glucose Metabolism and Other Markers of Metabolic Syndrome (Glucogold)	126	20–60-year-old patients with a BMI ≥ 25 suffering from pre-diabetes or early diagnosed diabetes	2016	Measured change in the oral disposition index and HbA1c	NA	84 days
117	NCT04149639	A Study Investigating the Effectiveness of a LifeSeasons NeuroQ Supplement with Lifestyle Changes to Improve Cognitive Function in Healthy Adults Who Have One or More Risk Factors for Cognitive Decline	40	≥45-year-old patients with risk factors for cognitive decline	2020	Measured change in cognition as assessed by the change in the Neurocognitive Index (NCI) score from the CNS-Vital Signs (CNS-VS) panel	NA	135 days
118	NCT01716637	Short Term Efficacy and Safety of Perispinal Administration of Etanercept in Mild to Moderate Alzheimer’s Disease	12	60–85-year-old patients with a diagnosis of Alzheimer’s disease	2016	Difference in the effects of the treatment for 6 weeks with etanercept + nutritional supplements versus nutritional supplements alone on the Mini-Mental Status Examination (MMSE) score	1	16 weeks
119	NCT01752868	Can Fish Oil and Phytochemical Supplements Mimic Anti-Aging Effects of Calorie Restriction?	56	40–60-year-old patients with a BMI of 21–30 kg/m^2^ who are sedentary to moderately active	2012	Carotid-femoral pulse wave velocity	NA	6 months
120	NCT00799630	Effects of Nutraperf Consumption in Runners	14	18–46-year-old healthy male distance runners	2008	Measurement of different metabolic parameters (heart rate, oxygen consumption, respiratory quotient, ventilation, glycemia, lactatemia) on central and peripheral fatigue and on cognitive parameters	NA	NA
121	NCT04765527	Turmeric and Exercise-Induced Muscle Damage and Oxinflammation	53	18–50-year-old healthy individuals who are willing to exercise	2021	Measuring a change in the serum concentration of creatine kinase	NA	4 days
122	NCT02413099	The Efficacy and Safety of New Herbal Formula (KBMSI-2) in the Treatment of Erectile Dysfunction	44	18–40-year-old male patients with a history of erectile dysfunction	2013	Measuring a change in the EF domain scores of the IIEF questionnaire from the baseline	4	8 weeks
123	NCT01906840	Role of Turmeric on Oxidative Modulation in ESRD Patients	48	≥18-year-old patients who undergo regular dialysis	2012	Measuring the effects of turmerics on oxidative stress markers	2	8 weeks
124	NCT01646047	Diabetes Visual Function Supplement Study (DiVFuSS)	70	≥18-year-old patients with a ≥5-year history of diabetes mellitus	2014	Measuring changes in visual function	NA	6 months
125	NCT02369536	Efficacy of a Natural Components Mixture in the Treatment of non-Alcoholic Fatty Liver Disease (NAFLD) (NUTRAFAST)	126	18–80-year-old patients with non-alcoholic fatty liver disease (NAFLD)	2016	Hematic levels of hepatic enzymes AST, ALT and GGT	NA	3 months
126	NCT02088307	Study of the Cardiovascular Vitamin, CardioLife	21	18–90-year-old patients with cardiovascular disease	2016	Change in blood pressure	NA	6 months
127	NCT05089318	Evaluation of Flexofytol^®^ PLUS in Hand Osteoarthritis.	239	≥45-year-old patients with hand arthritis and a regular use of analgesia	2021	Pain using a Visual Analog Scale (VAS)	NA	84 days
128	NCT03482401	Disposition of Dietary Polyphenols and Methylxanthines in Mammary Tissues from Breast Cancer Patients (POLYSEN)	40	≥18-year-old patients diagnosed with breast cancer	2019	Quantification of dietary polyphenols and methylxanthines in breast tissues	NA	24 months
129	NCT04890704	Curcuminoids and Contrast-induced Acute Kidney Injury	96	18–80-year-old patients undergoing elective CAG with a stable eGFR of 15–60 mL/min/1.72 m^2^	2019	The incidence of CI-AKI development between the addition of curcuminoids to the standard protocol and the standard protocol alone in patients who underwent CAG	1	48 h
130	NCT00219882	Safety Study of Orally Administered Curcuminoids in Adult Subjects with Cystic Fibrosis (SEER)	11	18–40-year-old patients who suffer from cystic fibrosis (homozygous for the ΔF508 CFTR genotype)	2006	Safety and tolerability of 14 days of treatment with orally administered curcuminoids, as assessed by adverse events, laboratory parameters and spirometry	1	14 days
131	NCT04844658	COVID-19, Hospitalized, PatIents, Nasafytol	51	≥18-year-old patients with a recent hospitalization due to SARS-CoV-2	2021	Improvement of the patient’s clinical condition based on the WHO ordinal outcomes score, the duration of hospitalization, mortality, fever, oxygen therapy, adverse events and several blood parameters	NA	14 days
132	NCT03065504	Turmeric and Turmeric-containing Tablets and Sebum Production	30	18–50-year-old healthy individuals	2017	Change in facial sebum production	NA	4 weeks
133	NCT04281758	Comparison of Plasma Caffeine Concentration After Oral Consumption of Caffeinated Beverages with Varied Bioactive Compounds in Healthy Volunteers	16	18–55-year-old healthy individuals willing to avoid caffeine and alcohol for a period of time	2020	Incremental area-under-the-concentration-curve (iAUC)	1	210 min
134	NCT04258501	Exploratory Study of Efficacy on Selected Natural Extracts Reducing Post Prandial Blood Glucose Response	72	20–50-year-old healthy individuals with a normal BMI	2012	Change in post-prandial blood glucose	NA	2 h

## 6. Limitations

In this paper, we have only focused on the key therapeutic activity of curcumin. Additionally, our focus has been on those activities of curcumin that are well characterized. Other aspects of curcumin activity, such as those associated with beneficial effects in neurological disorders, were not reviewed in this study. This alludes to the fact that such results have not been investigated in detail or explicated in the clinical trials. For example, for the phase 2 trial—curcumin in patients with mild to moderate Alzheimer’s disease—no results are posted in the trial database. This aspect may be attributed to the fact that the trial did not exhibit any beneficial outcome, more so because the delivery of curcumin across the blood-brain barrier has always been challenging. On the same note, we have not touched upon the aspect of curcumin delivery, as this is not only outside the scope of the review but also requires a detailed discussion which will make the present manuscript inadvertently lengthy. Readers are directed to some excellent reviews published in recent times for further details, if interested [295,296,297,298].

## 7. Conclusions and Future Directions

Curcumin is a pleiotropic molecule with a flexible structure with diverse biological functions. It is a potent proteasome inhibitor that increases the p53 level and induces apoptosis by mitochondrial caspase activation. Curcumin also disrupts 26S proteasome activity by inhibiting DYRK2 in different cancerous cells, resulting in the inhibition of cell proliferation [299]. However, further research is required to establish curcumin’s precise epigenetic regulatory effect for preventing and curing lethal diseases such as cancers. Curcumin may also act as an epigenetic regulator in neurological disorders, inflammation and diabetes. It can be effectively used as a histones modifier (acetylation/deacetylation), which is among the most important epigenetic changes responsible for gene expression alterations, leading to the modulation of the risks of rheumatoid arthritis and cancer.

Curcumin has shown therapeutic potential against several human diseases. The underlying mechanism for curcumin’s clinical efficacy seems to be the modulation of numerous signaling molecules. However, because of the complex nature of some diseases, the underlying mechanism in many cases remains unclear. Pharmacokinetic data indicate an almost 40-fold increase in blood levels in cases where curcumin was administered via formulation compared to pure form [300]. The poor bioavailability and limited adverse effects reported by some investigators are a major limitation to the therapeutic utility of curcumin. Nanocurcumin has shown a higher solubility and bioavailability in comparison to curcumin in recent studies [301]. Curcumin linked to phosphatidylcholine (which forms the fytosome–curcumin complex) has shown better bioavailability upon oral administration in rats [302]. We hope that the results from ongoing clinical trials will provide a deeper understanding of curcumin’s therapeutic potential and help to place this interesting molecule at the forefront of novel therapeutics.

The use of nanotechnology and a targeted drug delivery system has been shown to improve the cellular uptake, tissue specificity and effectiveness of curcumin. Although several nanosystems have been explored for the delivery of curcumin, due to its ability to inhibit the ABC efflux transporter [303], the combination nanoparticles of curcumin must be tested in cancerous cells once the proper dosage is determined. Most of the experiments using curcumin formulations have only been tested in pre-clinical models. The issue of cellular toxicity needs to be addressed by studying its activity in humans. Cost-effective techniques for curcumin nanoencapsulation are an emerging industrial requirement. The clinical trials to date have been conducted on a limited group of patients. Moreover, the tissue specificity of nanoparticles needs to be evaluated.

The combination of curcumin with other therapeutic reagents can further be explored. Case in point, a recent study showed that fecal microbial transplantation (FMT) leads to favorable outcomes in metabolic syndrome [304]. It would be interesting to appraise what happens in a group where a combined approach of FMT, fiber supplementation and curcumin is employed. For example, Liraglutide is used in the treatment of obesity because it induces weight loss; curcumin can be supplemented in a combined formulation with liraglutide to add to its benefits [305].

Currently, in our group, we are assessing the therapeutic potential of curcumin in combination with vitamin D and lipids of minor physiological abundance in attenuating inflammation in chondrocytes treated with LPS. The rationale behind using such a combination is that lipids [198] of minor physiological abundance, such as lysosulfatide (which is present in HDL particles) and vitamin D (which has a steroid nucleus), will augment the solubility and thus the bioavailability of curcumin [199].

## Figures and Tables

**Table 1:** Methods for curcumin extraction.

Method Name	Yield	Advantage(s)	Drawback(s)	Reference
**Microwave-assisted** **extraction (MAE)**	The yield obtained using MAE is 4.98%. The *Curcuma longa* plant was soaked in methanol and extracted using acetone via a dual heating mechanism under microwave energy.	Safe and cost-effective method. Retains the biological activity of the extracted compounds.	Time-consuming, as the vessel needs to be slowly cooled to avoid the loss of volatile components. Involves an initial high cost for setting up the equipment on a large scale.	[28]
**Enzyme-assisted ** **extraction (EAE)**	The yield obtained using EAE is 5.73%. Turmeric was pretreated using alpha-amylase and amyloglucosidase enzymes. Extraction was performed using N,N- dipropyl ammonium N,N’-dipropylcarbamate.	Mild reaction conditions are cost-effective, eco-friendly and feasible.	Low purity of the final product.	[29]
**Supercritical fluid ** **extraction (SFE)**	The yield obtained using SFE is 4.3%. A two-step extraction was performed using SFE, followed by pressurized liquid extraction using ethanol as solvent.	Short extraction time and mild operating temperature. The manufacturing costs are lower than those of conventional methods.	High technical complexity, and many operational parameters need to be optimized before the initiation of the extraction process.	[30]
**Ultrasound-assisted ** **extraction (UAE)**	The yield obtained using UAE is 1.03%. Optimal conditions were a 60% amplitude and a 3/1 (s/s) pulsed interval. Ethanol was the extraction solvent.	Greater solvent penetration into the samples increases the contact surface area, which increases efficiency.	Ultrasound can lead to the degradation of the final purified product.	[31]
**Ionic liquid-based ** **extraction (ILE)**	The yield obtained using ILE is 6.18%. The ionic liquid used for extraction was an anionic [Omin][Br-] aqueous solution.	Eco-friendly and increases extraction efficiency.	High cost involved in the preparation and use.	[32]

**Table 2:** Activity of curcumin against cancer.

Cancer Type	Curcumin Conc.	Signaling Molecules Up/Downregulated	Overview	Delivery Modes	Ref.
**Prostate cancer**	10–100 μM	Downregulates NF-κB, AP-1, Cyclin D1, CXCL-1 and CXCL-2, Bcl-2, Bcl-xL and XIAP.	-Curcumin is a potent inhibitor of NF-κB in both ADPC and AIPC cells, thereby preventing cell proliferation and inducing apoptosis. -Curcumin restores the response of AIPC cells to anti-androgen treatment. -Prevents metastasis in AIPC cells.	-Free curcumin or in combination with chemotherapeutic agents such as TRAIL. -Curcumin in poly(lactic-co-glycolic) acid. -Using the nanoparticle formulation of curcumin. -Curcumin-loaded liposomes.	[84,85,86,87,88,89,90,91,92,93,94,95]
**Breast ** **cancer**	10–40 μM	Downregulates Bcl-2, CXCL-1, CXCL-2, MMP-9, urokinase plasminogen activator, intercellular adhesion molecule 1 and chemokine receptor 4, PECAM-1, Cyclin D1 and p65.	-Curcumin can suppress ODC activity and inhibit cell proliferation. -Inhibits the MPA-induced secretion of pro-angiogenic factors such as VEGF.	-Hyaluronic acid-modified mesoporous silica nanoparticles. -Chitosan nanoparticles. -Zinc oxide nanoparticles. -In combination with niclosamide using PLGA nanoparticles. -PEGylated PLGA nanoparticles -Nanovesicles.	[21,96,97,98,99,100,101,102,103,104,105,106,107]
**Colon ** **cancer**	10–50 µM	Downregulates TNFα, JNK activation, miR-21 and COX-2.	-Curcumin inhibits the activation of the TLR4/MyD88/NF-κB signaling axis. -Reduces IκB kinase activity and inhibits the degradation of IkBα. -Inhibits the production of TNF-α, IL-6 and IL-12. -Inhibits Foxp3 expression and enhanced interferon-gamma secretion in regulatory T cells.	-Curcumin in silica nanoparticle. -Polymeric nanocarrier. -Curcumin-loaded thiolated chitosan nanoparticle. -Dendrosomal carrier. -Curcumin-loaded micelles. -Curcumin–PLGA nanoparticles.	[108,109,110,111,112,113,114,115,116,117,118,119]
**Pancreatic**	10–50 µM	Downregulates EGFR, COX-2, NF-κb, AKT and Prostaglandin E2.	-Inhibited cell survival and enhanced apoptosis in pancreatic adenocarcinoma cell lines. -Suppressed tumor growth by inhibiting the NF-KB pathway. -Induced apoptosis via ATM/Chk1. -Anti-proliferative activity by suppressing Sp1 and disrupting NF-κB translocation to the nucleus.	-Curcumin analogues PEGylated Curcumin, [Dlys6]-LHRH and its analog called L49H37. -CDF and PEGylated curcumin. -Liposomal Curcumin. -Curcumin analogues with the hydroxyl group. -Magnetic particles that were used to encapsulate curcumin. -Ester-mediated conjugations of curcumin to cholesteryl-hyaluronic acid nanogel.	[120,121,122,123,124,125,126,127,128,129,130,131]
**Gastric**	10–100 µM	Downregulates the Akt pathway, BCL-2, COX-2 and cyclin D1.	-Induced apoptosis by activating caspase-3,PARP; reduction in Bcl-XL levels. -Curcumin also activates the Fas pathway by stimulating the activity of caspase-8. -Activated Bax protein expression and inhibited the Bcl-2 protein -Suppressed the transition of the cancer cells from the G(1) to S phase.	-Cyclodextrin complexes with curcumin. -Nanoparticles such as polymer-encapsulated ZnO nanoparticles. -Microsponges using polymers such as ethyl cellulose and polyvinyl alcohol. -Curcumin-loaded nanoemulsion. -Cationic polysaccharides such as chitosan.	[132,133,134,135,136,137,138,139,140,141,142]
**Lung**	5–50 µM	Downregulates the Akt pathway, BRCA pathway, Beta-catenin signaling and MMP-2 and upregulates caspase-3, Bax and p53.	Induces apoptotic cell death by activating caspase-7 and 3. -Enhances PARP cleavage and stimulates ER stress. -Enhances ROS production to cause apoptosis. -Increases the sensitivity of cancer cells to chemotherapy. -Induces DNA damage and prevents the migration of cancer cells.	-Lipid-based liposome. -Polymeric carrier and micelle. -Chitosan microsphere. -Polymeric and lipid nanoparticle. -Nanocrystal.	[143,144,145,146,147,148,149,150,151,152,153,154]
**Oral**	10–100 µM	-Prevents cell proliferation and promotes apoptosis.	-Curcumin reduces the migration and progression of TSCC cells, promotes apoptosis and inhibits tumorigenesis. -Suppresses the CAF (cancer-associated fibroblast)-mediated proliferation and tumorigenicity of Cal27 by inhibiting TSCC CAFs.	-Nanohybrid formulation. -Lozenges. -Silica nanoparticles. -Mucoadhesive nanogel system.	[155,156,157,158,159,160]
**Skin**	10–50µM	-Shows effective anti-proliferative activity.	-Antiproliferative effect, as they effectively inhibit the clonogenic ability in melanoma cells.	-Cationic liposomes. -Ethosomal nanocarriers.	[161,162]

**Table 4:** Effect of curcumin on gut microbiota and its mechanism of action.

Curcumin Doses	Effect on Gut Microbiota	Molecular Mechanisms	Model	Ref.
**Curcumin at a low dose (1 g/day)**	Curcumin shifted the structure of gut microbiota	Curcumin enervated the Western diet-induced development of atherosclerosis and type 2 diabetes mellitus	Sprague Dawley rats	[225]
**100 mg/kg/day**	Lowers the increasing abundance of the genera *Anaerotruncus* and *Helicobacter* in the gut microbiota	Decreases the estrogen level, resulting in an increase in body weight	Wistar rats	[226]
**100 mg/kg/day**	Curcumin affected the presence of *Prevotellaceae, Bacteroidacea, and Rikenellaceae* in gut microbiota	Curcumin possesses anticancer activity in vitro and in preclinical animal models via the activation of caspases 3, 8 and 9 in the colon cancer cell lines	Fecal sample	[227]
**8000 mg per day**	Increase in *Lactobacillus* and decrease in *Coriobacterales*	Induction of apoptosis through the COX-2 and non-COX-2 pathways. It targets cancer stem cells (CSC) through direct or indirect influences on the CSC self-renewal pathways.	Colon cancer cell lines, SW480 and SW62	[228]
**0.2% (*w*/*w*) nanoparticles of curcumin**	Increase in butyrate-producing bacteria and the fecal butyrate level	Mucosal mRNA expression of inflammatory mediators and the activation of NF-κB in colonic epithelial cells were suppressed by curcumin nanoparticles	BALB/c mice	[229]

## Abbreviations

ACE	Acetyl coenzyme
ADPC	Androgen-dependant prostate cancer
AIPC	Androgen-independent prostate cancer
AKT	Protein kinase B (also called PKB)
ALT	Alanine transaminase
AMPK	AMP-activated protein kinase
AP	Activator protein
AST	Aspartate aminotransferase
BAFF	B cell activating factor
BCL	B cell lymphocyte
Bcl	B cell lymphoma
Bcl-xL	B cell lymphoma-extra large
CD	Cluster differentiation
CHOP	Cytoxan hydroxydaunorubicin oncovin prednisone
Coll2	Collagen
COX	Cyclooxygenase
Cur-CQD	Curcumin carbon quantom dots
CXCL	Chemokine (C-X-C motif) ligand
DHA	Docosahexaenoic acid
DYRK	Dual specificity tyrosine phosphorylation-regulated kinase
EAE	Enzyme-assisted extraction
EBP	Enhancer binding protein
EPA	Eicosapentaenoic acid
ERK	Extracellular-regulated kinase
ERK	Extracellular-regulated kinase
FFA	Free fatty acid
FOX	Forkhead box protein
FtsZ	Filamenting temperature sensitive mutant Z
GST	Glutathione S- transferase
Hp	Haptoglobin
HSP	Heat shock protein
IFN	Interferon
IKBα	Inhibitor of kappa light chain gene enhancer in B cells
IL	Interlekin
ILE	Ionic liquid-based extraction
IL	Interleukin
iNOS	Inducible nitric oxide syntase
JAK/STAT	Janus kinase/signal transducers and activators of transcription
JNK	Jun N-terminal kinase
LPS	Lipopolysaccharides
MAE	Microwave-assisted extraction
MAPK	Mitogen-activated protein kinase
MCP	Methyl-accepting chemotaxis protein
M-CSF	Macrophage colony stimulating factor
MDA	Malondialdehyde
MIC	Minimum inhibitory concentration
MIP	Macrophage inflammatory protein
MMP	Matrix metalloproteinase
MPA	Medroxyprogesterone acetate
MTOR	Mammalian target of rapamycin
MyD	Myeloid differentiation
NF-κkB	Nuclear factor kappa-light-chain-enhancer of activated B cells
NLRP	Nod-like receptor protein
NOD	Nucleotide oligomerization domain
Nrf	Nuclear respiratory factor
NSAID	Non-steroidal anti-inflammatory drugs
OC	Oleocanthal
ODC	Ornithine decarboxylase
PECAM	Platelet endothelial cell adhesion molecule
PLGA	Poly(D,L-Lactic-co-glycolic acid)
PPAR	Peroxisome proliferator-activated receptors
Rac 1	Rass-related C 3 botulinum toxin substrate 1
RANKL	Receptor activator of nuclear factor kappa B ligand
RANTES	Regulated on activation, normal T cell expressed and secreted
RNS	Reactive nitrogen species
ROS	Reactive oxygen species
SDH	Succinate dehydrogenase
SFE	Supercritical fluid extraction
Shh	Sonic hedgehog protein
SOD	Superoxide dismutase
STZ	Streptozotocin
TBARs	Thiobarbituric acid reactive substances
TLR	Toll-like receptor
TLR	Toll-like Receptor
TNF	Tumor Necrosis Factor
TRAIL	Tumor necrosis factor (TNF)-related apoptosis-inducing ligand
TRAIL	Tumor necrosis factor-related apoptosis-inducing ligand
UAE	Ultrasound-assisted extraction
UPR	Unfolded protein response
VEGF	Vascular endothelial growth factor
Wnt	Wingless related integration site
XIAP	X-chromosome-linked inhibitor of apoptosis protein
ADPC	Androgen-dependant prostate cancer
AIPC	Androgen-independent prostate cancer
AP	Activator protein
Bcl	B cell lymphoma
Bcl-xL	B cell lymphoma-extra large
COX	Cyclooxygenase
CXCL	Chemokine (C-X-C motif) ligand
FOX	Forkhead box protein
IL	Interleukin
JNK	Jun N-terminal kinase
MMP	Matrix metalloproteinase
MPA	Medroxyprogesterone acetate
NF-kB	Nuclear factor kappa-light-chain-enhancer of activated B cells
ODC	Ornithine decarboxylase
PECAM	Platelet endothelial cell adhesion molecule
PLGA	Poly(D,L-Lactic-co-glycolic acid)
TLR	Toll-like receptor
TNF	Tumor necrosis factor
TRAIL	Tumor necrosis factor-related apoptosis-inducing ligand
TRAIL	Tumor necrosis factor (TNF)-related apoptosis-inducing ligand
VEGF	Vascular endothelial growth factor
XIAP	X-chromosome-linked inhibitor of apoptosis protein
